# eIF4F controls ERK MAPK signaling in melanomas with *BRAF* and *NRAS* mutations

**DOI:** 10.1073/pnas.2321305121

**Published:** 2024-10-22

**Authors:** Barbora Valcikova, Natalia Vadovicova, Karolina Smolkova, Magdalena Zacpalova, Pavel Krejci, Shannon Lee, Jens Rauch, Walter Kolch, Alexander von Kriegsheim, Anna Dorotikova, Zdenek Andrysik, Rachel Vichova, Ondrej Vacek, Karel Soucek, Stjepan Uldrijan

**Affiliations:** ^a^Department of Biology, Faculty of Medicine, Masaryk University, Brno 62500, Czech Republic; ^b^International Clinical Research Center, St. Anne’s University Hospital, Brno 60200, Czech Republic; ^c^Laboratory of Cell Signaling, Institute of Animal Physiology and Genetics of the Czech Academy of Sciences, Brno 60200, Czech Republic; ^d^Systems Biology Ireland, School of Medicine, University College Dublin, Dublin D04 V1W8, Ireland; ^e^School of Biomolecular and Biomedical Science, University College Dublin, Dublin D04 V1W8, Ireland; ^f^Conway Institute of Biomolecular and Biomedical Research, University College Dublin, Dublin D04 V1W8, Ireland; ^g^Cancer Research UK Scotland Centre, Institute of Genetics and Cancer, University of Edinburgh, Edinburgh EH4 2XR, United Kingdom; ^h^Department of Pharmacology, School of Medicine, University of Colorado Anschutz Medical Campus, Aurora, CO 80045; ^i^Department of Cytokinetics, Institute of Biophysics of the Czech Academy of Sciences, Brno 61200, Czech Republic; ^j^Department of Experimental Biology, Faculty of Science, Masaryk University, Brno 62500, Czech Republic

**Keywords:** melanoma, ERK, MAP kinase, eIF4F, DUSP6

## Abstract

The eIF4F translation initiation complex plays a critical role in melanoma resistance to clinical BRAF and MEK inhibitors. In this study, we uncover a function of eIF4F in the negative regulation of the rat sarcoma (RAS)/rapidly accelerated fibrosarcoma (RAF)/mitogen-activated protein kinase kinase (MEK)/extracellular signal-regulated kinase (ERK) mitogen-activated protein kinase (MAPK) signaling pathway. We demonstrate that eIF4F is essential for controlling ERK signaling intensity in treatment-naïve melanoma cells harboring *BRAF* or *NRAS* mutations. Specifically, the dual-specificity phosphatase DUSP6/MKP3, which acts as a negative feedback regulator of ERK activity, requires continuous production in an eIF4F-dependent manner to limit excessive ERK signaling driven by oncogenic RAF/RAS mutations. Treatment with small-molecule eIF4F inhibitors disrupts the negative feedback control of MAPK signaling, leading to ERK hyperactivation and EGR1 overexpression in melanoma cells in vitro and in vivo. Furthermore, our quantitative analyses reveal a high spare signaling capacity in the ERK pathway, suggesting that eIF4F-dependent feedback keeps the majority of ERK molecules inactive under normal conditions. Overall, our findings highlight the crucial role of eIF4F in regulating ERK signaling flux and suggest that pharmacological eIF4F inhibitors can disrupt the negative feedback control of MAPK activity in melanomas with *BRAF* and *NRAS* activating mutations.

## Significance

Oncogenic signaling pathways are intricately regulated networks where alterations can lead to malignant progression but also provide opportunities for targeted interventions. This study uncovers an essential interplay between the eIF4F translation initiation complex, which contributes to melanoma resistance, and the extracellular signal-regulated kinase (ERK) signaling pathway, the primary therapeutic target in melanomas with *BRAF* and *NRAS* mutations.

The extracellular signal-regulated kinase (ERK) signal transduction pathway, which regulates cell proliferation, differentiation, and survival in response to extracellular stimuli, is constitutively activated in most malignant melanomas due to oncogenic mutations of *NRAS* or *BRAF* genes (1). While BRAF and MEK inhibitors can be effective in melanoma patients, resistance to these therapies often develops (2). The *NRAS*-driven melanomas pose a particular challenge as they cannot be treated by the combinations of BRAF and MEK inhibitors.

Recent findings suggest that melanoma cells with oncogenic mutations in the ERK pathway adjust the available feedback mechanisms to establish an ERK activity optimum, promoting maximal cancer cell growth and proliferation. Specifically, melanoma cells bearing the most common oncogenic mutation BRAF^V600E^ rely, at least partly, on the activity of the dual-specificity phosphatase DUSP6/MKP3, a negative feedback regulator of ERK signaling, to keep the ERK activity within the optimal range (3). Importantly, this pathway is rewired, and a new optimum is established in *BRAF*-mutated melanoma cells in response to therapy with BRAF or MEK inhibitors (4, 5). Cells emerging as therapy-resistant can even become addicted to the BRAF inhibitors, and acute withdrawal of the drug can overactivate the ERK pathway and lead to cell cycle arrest and cell death (6, 7). These results suggest that there is an ERK mitogen-activated protein kinase (MAPK) fitness zone and hyperactivation of the ERK pathway in melanoma cells is not tolerated. In another study, ERK2 overexpression also promoted melanoma cell death (8). However, the ERK activity optimum in melanoma remains to be characterized, partly due to the lack of tools to hyperactivate ERK signaling effectively.

The eukaryotic translation initiation factor eIF4F is a promising therapeutic target in various human cancers (9). In metastatic melanoma, eIF4F has been identified as a driver of resistance to therapies targeting BRAF and MEK, and simultaneous inhibition of BRAF and eIF4F activity synergized in killing tumor cells (10). A compound disrupting the eIF4F complex attenuated the growth of BRAF inhibitor-resistant melanomas (11). Moreover, a combination of an eIF4F inhibitor with vemurafenib and cobimetinib prevented the emergence of persister melanoma cells that can tolerate the exposure to lethal concentrations of the BRAF and MEK inhibitors (12). In the context of *NRAS*-mutant melanoma, the resistance to MEK inhibition was also associated with the persistent formation of the active eIF4F complex (13). These findings indicate a therapeutic potential for eIF4F inhibitors in melanoma. However, the molecular mechanisms contributing to eIF4F-mediated resistance to BRAF and MEK inhibitors have not been fully characterized.

In the current study, we used specific small-molecule eIF4F complex inhibitors as chemical probes to study the cross talk between eIF4F and the ERK pathway in melanoma cells. We provide evidence that this cross talk is not limited only to cells resistant to drugs targeting BRAF and MEK kinases. Instead, eIF4F activity is essential for controlling ERK MAPK signaling flux in treatment-naïve human melanoma cells bearing *NRAS* and *BRAF* oncogenic mutations.

## Results

### eIF4F Activity Controls Steady-State Protein Synthesis in Human Melanoma Cells.

Initially, we weighed up the possibility of using the CRISPR–Cas9 genetic approach to study the role of eIF4F in the biology of treatment-naïve melanoma cells. We started by analyzing the results of genome-wide CRISPR–Cas9 screens in human cancer cell lines from various cancer types performed by Behan et al. (14) available at the Project Score database (15). The analysis revealed that the genetic disruption of the *EIF4A1*, *EIF4E*, and *EIF4G1* genes encoding the eIF4A, eIF4E, and eIF4G subunits decreased fitness of most melanoma cell lines, arguing against the use of knock-out cell lines to study the impact of eIF4F inhibition in melanoma (*SI Appendix*, Fig. S1). Therefore, we chose the chemical biology approach for our study, taking advantage of well-characterized and selective small-molecule compounds targeting eIF4F (eIF4Fi).

**Fig. 1.**
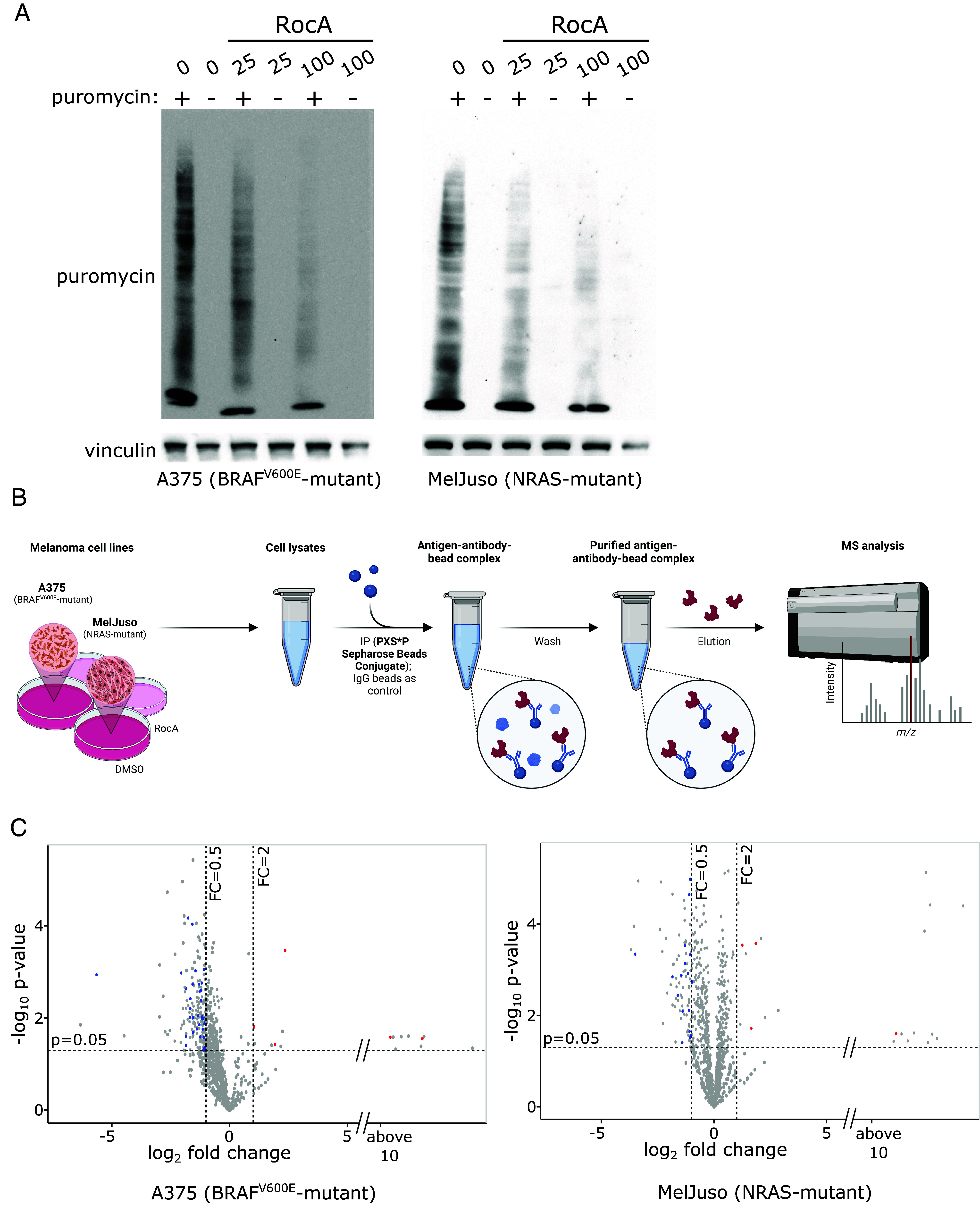
In melanoma cells, a proportion of MAPK targets are synthesized in an eIF4F-dependent manner. (*A*) The effect of eIF4F inhibition on the rate of protein synthesis in melanoma cells was analyzed using the puromycylation assay. A375 (BRAF^V600E^) and MelJuso (*NRAS*-mutant) cells were treated with the indicated concentrations of Rocaglamide A (RocA) for 4 h. Next, newly synthesized peptide chains were covalently labeled by adding puromycin (90 µM final concentration) to the cell cultures for 15 min, followed by cell lysis, SDS-PAGE, and Western blotting. Vinculin served as a loading control. The controls were treated with an equivalent volume of the vehicle (DMSO). (*B*) Schematic representation of the identification of MAPK targets responding to eIF4F inhibition. A375 and MelJuso cells were treated with the eIF4F inhibitor Rocaglamide A (RocA, 100 nM) for 20 h. Phospho-MAPK/CDK Substrate Motif (PXS*P and S*PXK/R) Kit was used for the immunoprecipitation of cell lysates, followed by the LC-MS/MS analysis to identify potential MAPK targets affected by eIF4F inhibition. Created with BioRender.com. (*C*) A proportion of MAPK targets is downregulated in response to eIF4F inhibition in both melanoma subtypes. Volcano plots depict changes in protein levels in cells treated with Rocaglamide A (RocA, 100 nM) for 20 h. Previously identified ERK targets among the downregulated proteins are depicted in blue and characterized by log2 Fold Change < −0.5 and *P*-value < 0.05. Red dots represent up-regulated previously identified ERK targets (http://sys-bio.net/erk_targets/targets_all.html), characterized by log2 Fold Change > 2 and *P*-value < 0.05.

Flavaglines, also known as rocaglates, rank among the best-characterized and most selective inhibitors of the eIF4F complex. Rocaglamide A (RocA), cyclopenta[b]benzofuran found in the medicinal plants of the genus *Aglaia*, inhibits the eIF4A helicase subunit of the eIF4F complex and sequesters eIF4A onto mRNA, converting it into a sequence-selective translation repressor (16, 17). First, we analyzed the impact of this compound on the rate of protein synthesis in a human melanoma cell line representing the most common melanoma genetic subtype (A375, BRAF^V600E^ mutation). Cells were treated for 4 h with RocA at 25 and 100 nM concentrations, and newly synthesized polypeptides were labeled for 15 min with puromycin. Western blot detection of puromycylated nascent polypeptide chains showed that RocA potently inhibited protein synthesis (Fig. 1*A*), indicating that a significant proportion of proteins synthesized in growing melanoma cells were produced in an eIF4F-dependent manner. A similar observation was made with MelJuso cells representing the second-most common genetic subtype of human melanoma bearing an oncogenic *NRAS* mutation (Fig. 1*A*). Next, we assessed the potential antiproliferative and cytotoxic effects of RocA treatments. Results of the 48 h MTT assays indicated that the compound affected cell proliferation at a range of concentrations (*SI Appendix*, Fig. S2*A*). Taking the potential limitations of the MTT assay into consideration, we decided to determine the growth of melanoma cell cultures also using cell staining. The results showed that both *BRAF*- and *NRAS*-mutant melanoma cultures were viable after 24 h treatments despite the significant impact of RocA on protein synthesis (*SI Appendix*, Fig. S2 *B* and *C*).

### eIF4F Inhibition Impacts on ERK MAPK Targets in Melanoma Cells.

Enhanced eIF4F-mediated translation was suggested to partly compensate for lower ERK activity in BRAF and MEK inhibitor-treated melanoma cells (10). This result indicated that enhanced eIF4F activity could promote the expression of ERK pathway targets contributing to the survival, growth, and proliferation of BRAF/MEK inhibitor-treated cells. Considering the potent downregulation of protein synthesis by RocA observed in Fig. 1*A*, we expected that eIF4F inhibition could have a negative impact on the expression of ERK targets. We chose a proteomic approach to assess the proportion of MAPK kinase substrates synthesized in an eIF4F-dependent manner in human *BRAF*- and *NRAS*-mutant melanoma cells. We treated the A375 and MelJuso cell lines with 100 nM RocA for 20 h, followed by cell lysis and immunoprecipitation of the lysates using PTMScan® Phospho-MAPK/CDK Substrate Motif (PXS*P and S*PXK/R) Kit and LC-MS/MS analysis of enriched peptides (Fig. 1*B*). Then we took advantage of the online database Compendium of ERK targets (18) to assign the previously identified ERK kinase targets. Results presented in Fig. 1*C* and Datasets S1 and S2 indicate that a proportion of ERK kinase targets might be downregulated in melanoma cells upon eIF4F inhibition.

However, a small subset of known and potential MAPK targets identified in the mass spectrometry analysis appeared to be up-regulated in response to RocA treatment in melanoma cells. Considering that we analyzed changes in the levels of proteins precipitated by an antibody recognizing the phosphorylated MAPK target sequence, the observed upregulation could have two possible causes. First, it could be caused by an enhanced protein expression of some MAPK targets in response to RocA. The second, and more intriguing possibility could be the upregulation of MAPK activity in melanoma cells treated with eIF4Fi. In any case, these unexpected findings hinted at the possibility that the eIF4F action in the control of the oncogenic MAPK signaling in human melanoma might not be limited to the context of cellular resistance to BRAF and MEK inhibitors, warranting further studies in treatment-naïve melanoma cells.

### eIF4F Inhibition Promotes ERK Hyperactivation in Melanoma.

**Fig. 2.**
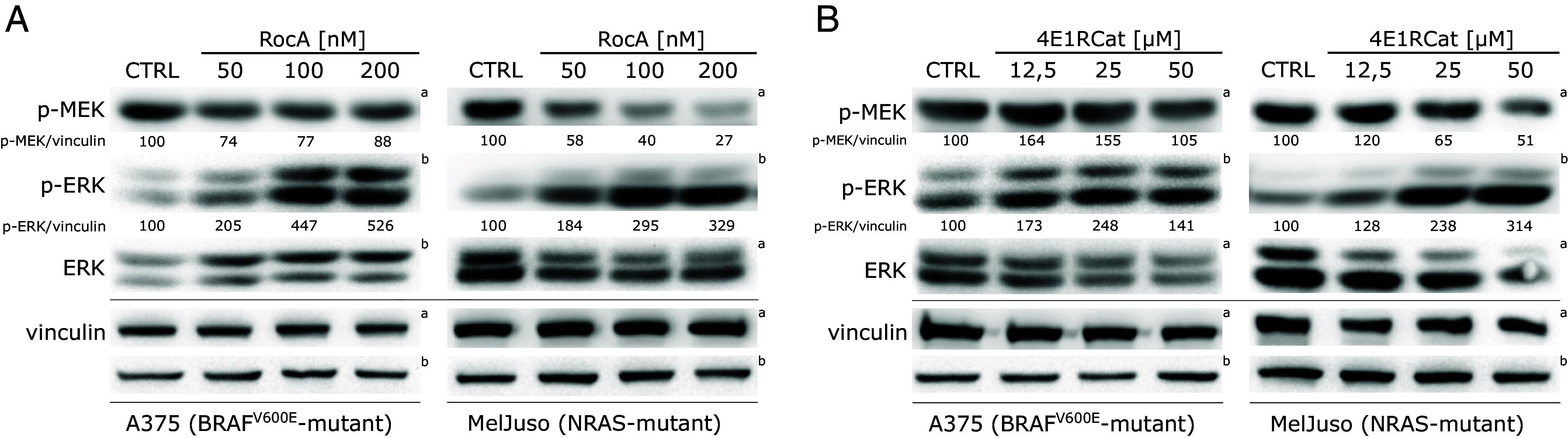
eIF4F inhibition promotes ERK activation in melanoma cells. Western blot analysis of A375 and MelJuso cells after 20 h treatment with increasing concentrations of small-molecule eIF4F inhibitors. (*A*) eIF4A inhibitor Rocaglamide A (RocA) or (*B*) eIF4E-eIF4G disruptor, 4E1RCat. The activity of MEK and ERK is represented by the ratio of phospho-MEK and phospho-ERK (p-MEK, p-ERK) to vinculin levels. Vinculin served as a loading control. The control samples (CTRL) were treated with an equivalent volume of the vehicle (DMSO). The upper index letters refer to the corresponding loading control detected on the same membrane.

To determine the potential impact of eIF4F inhibition on the ERK MAPK signaling pathway activity in melanoma, we analyzed the levels of active MEK and ERK kinases in RocA-treated A375 and MelJuso cells by SDS-PAGE and Western blotting. We observed highly increased p-ERK levels in response to a 20 h treatment with the eIF4A helicase inhibitor in both melanoma subtypes (Fig. 2*A*), despite the presence of strong oncogenic *BRAF* and *NRAS* mutations already highly activating the ERK pathway in these cells. A similar level of ERK hyperactivation in both melanoma subtypes was observed also in response to a structurally unrelated small-molecule inhibitor 4E1RCat, which blocks the eIF4F complex activity by disrupting the interaction of the eIF4G and eIF4E subunits (Fig. 2*B*). Surprisingly, while both inhibitors strongly enhanced the ERK activity, upregulation of the phosphorylation of its upstream activating kinase MEK was not observed in most cases (Fig. 2). These results suggested that the MAPK pathway signaling flux initiated by the oncogenic *BRAF* and *NRAS* mutations remained essentially unchanged at the level of RAF and MEK kinases. However, it was potently enhanced downstream of MEK at the level of the ERK kinase in eIF4Fi-treated melanoma cells, possibly due to a disruption of a negative regulatory mechanism.

Active MAPK signaling can modulate eIF4F activity via the ERK/MNK axis, promoting eIF4E phosphorylation at Ser209 (19–22). Although we assumed that highly active ERK signaling driven by oncogenic mutations might already fully stimulate eIF4E phosphorylation, we tested the possibility that the increase in ERK activity after eIF4Fi action could still lead to a rise in p-eIF4E levels. In both A375 and MelJuso cell lines, we observed a slight but distinct increase in the phosphorylation signal after RocA treatment (*SI Appendix*, Fig. S3). This indicated that, despite the oncogenic mutations in the ERK pathway in melanoma cells, there is still spare capacity for further increase of eIF4E phosphorylation. No significant increase in S209 phosphorylation was observed in 4E1RCat-treated cells, which could be explained by the different mechanisms of action of the two compounds. While RocA inhibits eIF4A and sequesters it on mRNAs, 4E1RCat disrupts the interaction between eIF4G and eIF4E. As eIF4G was shown to recruit MNK into the eIF4F complex (23), the increase in eIF4E phosphorylation may not be possible in 4E1RCat-treated cells. In any case, the increase in eIF4E phosphorylation might not have a significant impact on overall eIF4F activity in RocA-treated cells, as the inhibitory effect of rocaglates on translation initiation was shown to be independent of MNK activity and eIF4E phosphorylation (24).

### eIF4F Activity Is Required to Maintain DUSP6/MKP3 Expression in Melanoma Cells.

**Fig. 3.**
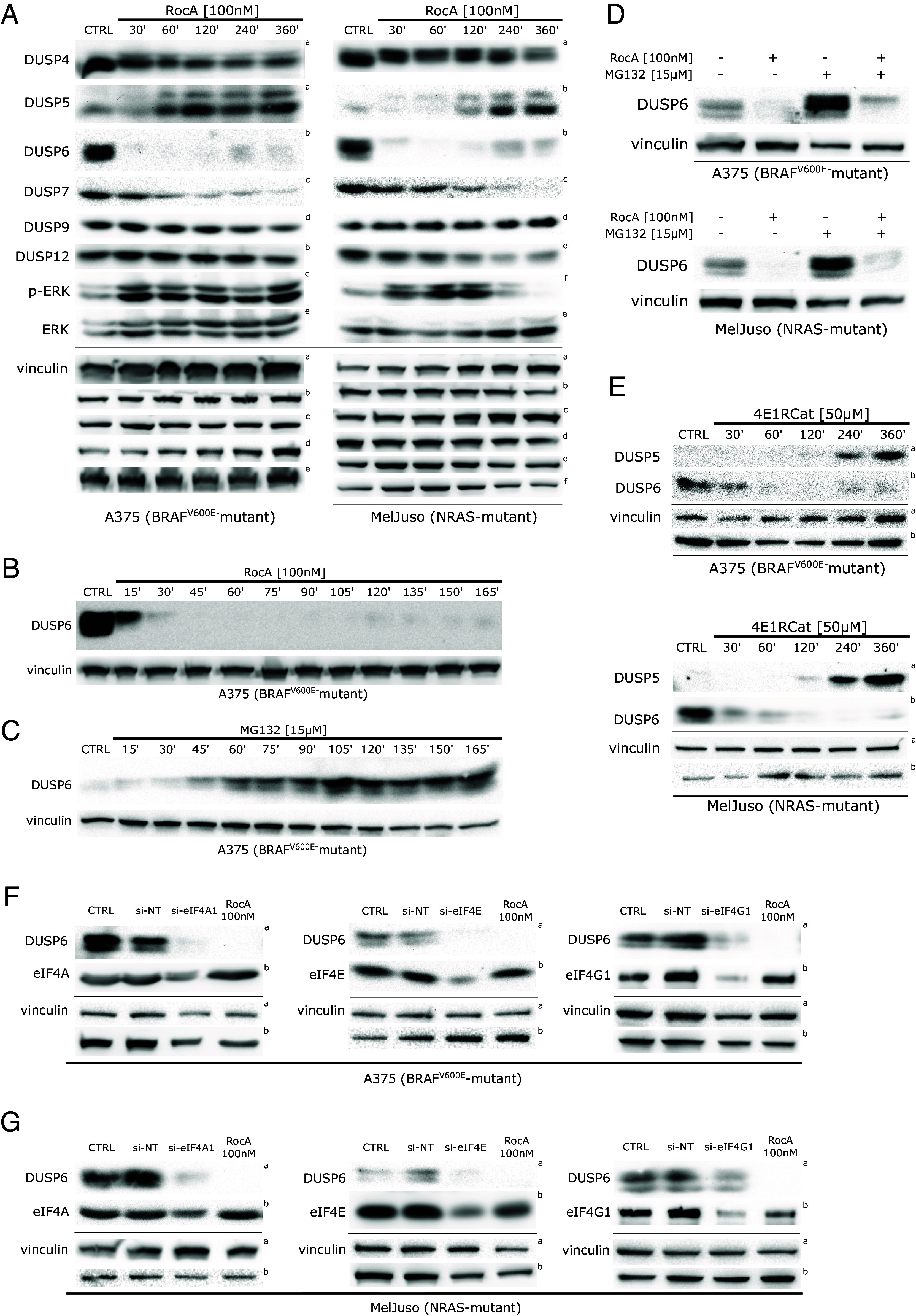
ERK activity in melanoma is limited by eIF4F-dependent expression of DUSP6/MKP3. (*A*) eIF4A inhibition induces dynamic changes in the levels of the DUSP/MKP family members, which function as critical negative regulators of the ERK pathway. Western blot analysis of A375 and MelJuso cells treated with RocA for indicated time periods. The upper index letters refer to the corresponding loading control detected on the same membrane. (*B*) Western blot analysis of A375 cells treated with Rocaglamide A (RocA) in shorter time periods shows the rapid degradation of DUSP6/MKP3 in response to eIF4F inhibition. (*C*) Western blot analysis of A375 cells treated with 15 µM MG132 showing the accumulation of DUSP6/MKP3 in response to proteasome inhibition. (*D*) The addition of a proteasome inhibitor led to a partial recovery of DUSP6/MKP3 levels caused by eIF4F inhibition alone, as shown in Western blot analysis of A375 and MelJuso cells treated with Rocaglamide A and/or MG132 for 16 h. (*E*) A structurally unrelated small-molecule eIF4Fi also promoted dynamic changes in the levels of DUSP6 and DUSP5. Western blot analysis was performed on lysates of A375 and MelJuso cells treated with 50 µM 4E1RCat for indicated periods. The upper index letters refer to the corresponding loading control detected on the same membrane. Vinculin served as a loading control. The control samples (CTRL) were treated with an equivalent volume of the vehicle (DMSO). (*F* and *G*) Knockdown of individual eIF4F subunits recapitulates the impact of small-molecule eIF4F inhibitors on DUSP6 expression in melanoma cells. Western blot analysis of A375 (*F*) and MelJuso (*G*) cells transiently transfected with siRNAs specific for eIF4A1, eIF4E, and eIF4G1 for 48 h. Non-targeting siRNAs (si-NT) were transfected in parallel as negative controls. 24 h treatment with RocA served as a positive control, while the control samples (CTRL) were treated with an equivalent volume of the vehicle (DMSO). The upper index letters refer to the corresponding loading control detected on the same membrane.

The best-characterized ERK negative regulators are the MAP kinase phosphatases (MKPs), also known as dual-specificity phosphatases (DUSPs). Some members of this enzyme family specifically target only one MAP kinase, while other can inhibit multiple MAPKs (25, 26). The expression of the ERK-specific phosphatases DUSP5 and DUSP6/MKP3 is positively regulated by active ERK, creating autoregulatory feedback loops limiting excessive ERK activation. In BRAF^V600E^ melanoma cells, DUSP6/MKP3 proved essential for controlling the oncogenic ERK signaling (3). We hypothesized that one or more MKPs negatively regulating ERK activity in melanoma cells could be expressed in an eIF4F-dependent manner. Simultaneously, if its protein half-life were sufficiently short, its depletion in response to eIF4F inhibition could lead to the observed potentiation of ERK activity. That is why we analyzed the changes of selected MKPs/DUSPs upon shorter (30 to 360 min) RocA treatments in MelJuso and A375 cells. Using Western blotting, we detected a rapid decline in the levels of three MAP kinase phosphatases capable of targeting active ERK (DUSP4, DUSP6/MKP3, and DUSP7), while changes in the levels of DUSP9 and DUSP12 were much less pronounced (Fig. 3*A*). Interestingly, at later time points (beyond 60 min RocA treatment) we detected an increase in the expression of the ERK-regulated phosphatase DUSP5, suggesting that the enhanced DUSP5 expression might constitute an additional feedback mechanism partly compensating for the lower levels of other DUSPs. Another interesting observation was a slight shift of DUSP4 toward higher molecular weight, indicating a potential induction of DUSP4 posttranslational modifications in response to eIF4Fi.

The rapid loss of DUSP6 expression correlated with an increase in p-ERK levels in both melanoma genetic subtypes (Fig. 3*A*). While in A375 cells, the increase remained present for the whole observed period, in MelJuso cells, the p-ERK levels went down again at later time points, possibly due to the activity of DUSP5. This result indicates the possibility of ERK activity oscillations in eIF4Fi-treated cells due to changing levels of DUSPs regulated by ERK but insensitive to eIF4F inhibition, such as DUSP5. The insensitivity of *DUSP5* transcript to eIF4Fi is probably linked to the short 5′ UTR compared to DUSP6 and its lower probability of forming complex secondary structures (*SI Appendix*, Fig. S4 *A* and *B*), as predicted by the MaxExpect online tool incorporated in the Predict a Secondary Structure Web Server v. 6.0.1 (https://rna.urmc.rochester.edu) (27).

In the next set of experiments, we concentrated on DUSP6/MKP3 as its rapid and profound decrease was the most prominent change among the analyzed DUSPs. Moreover, its previously established role in the negative control of ERK signaling in BRAF^V600E^ as well as *NRAS*-mutant melanoma cells (3, 28) suggested that DUSP6 loss could drive the observed ERK hyperactivation. The subsequent analyses confirmed that DUSP6/MKP3 was highly unstable in RocA-treated A375 melanoma cells, with a protein half-life of less than 15 min (Fig. 3*B*). Such instability indicated the presence of a molecular mechanism responsible for rapid, active DUSP6/MKP3 degradation in melanoma cells. To test the possible involvement of ubiquitin-mediated proteasomal degradation, we treated A375 melanoma cells with a small-molecule proteasome inhibitor MG132. As expected, the compound promoted a significant accumulation of the dual-specificity phosphatase (Fig. 3*C*). Importantly, in a different experiment, a cotreatment with MG132 at least partly rescued the eIF4Fi-induced DUSP6/MKP3 depletion in both melanoma subtypes (Fig. 3*D*).

To exclude the possibility of a previously unidentified eIF4F-independent off-target effect of RocA on DUSP6 protein stability, we treated melanoma cells with 4E1RCat, a structurally unrelated compound inhibiting eIF4F by a different mechanism. Crucially, we observed a rapid downregulation of DUSP6, followed by DUSP5 induction at later time points, in both melanoma genetic subtypes (Fig. 3*E*), confirming the observations made with RocA. Another independent confirmation of eIF4F-dependent regulation of DUSP6 levels in melanoma cells was provided by transient transfection of siRNAs specific for the *eIF4A1*, *eIF4G1*, and *eIF4E* transcripts. The knockdown of individual eIF4F subunits caused a significant drop in DUSP6 levels in both cell lines (Fig. 3 *F* and *G*). As expected, the siRNA-mediated knockdown of eIF4F subunits also promoted an increase in p-ERK levels in A375 melanoma cells (*SI Appendix*, Fig. S5). Collectively, the data indicated that eIF4F contributes to the control of ERK activity in melanoma cells by maintaining the continuous production of DUSP6 MAP kinase phosphatase.

### *EGR1* Transcript Is Up-Regulated in eIF4F Inhibitor-Treated Melanoma Cells.

**Fig. 4.**
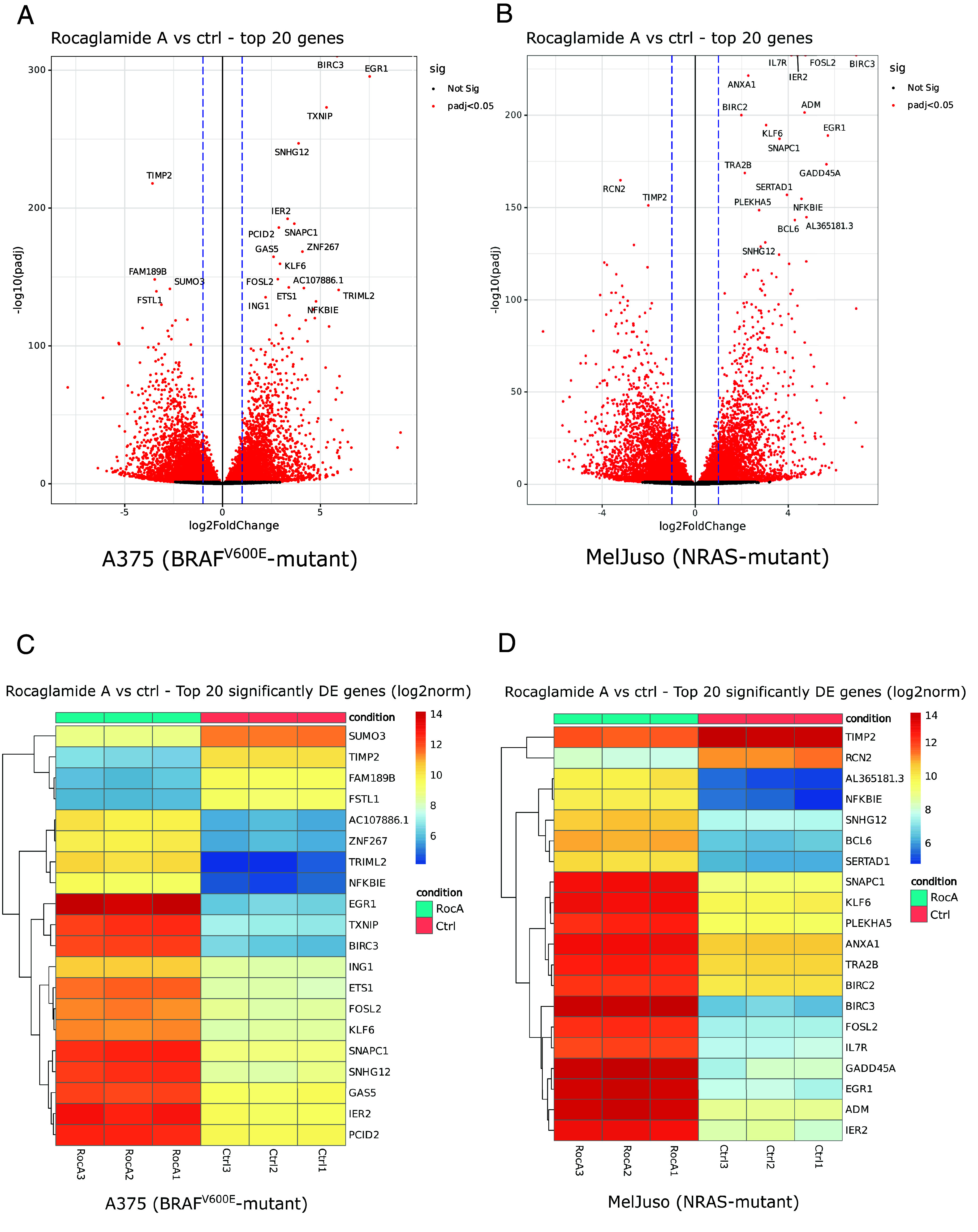
Changes in melanoma cell transcriptome in response to eIF4F inhibition. We used next-generation RNA sequencing to analyze transcriptome changes in response to 20 h RocA treatments. Volcano plots show overall changes in gene expression in A375 (*A*) and MelJuso (*B*) melanoma cells in response to eIF4Fi. Heat maps present the response to eIF4Fi of the top 20 differentially expressed genes in the individual independent biological replicates [A375 (*C*), MelJuso (*D*)].

Next, we decided to characterize the MAPK optimum in melanoma by assessing the extent of ERK activity changes in eIF4Fi-treated cells more quantitatively. This required a sensitive reporter system faithfully reflecting the ERK activity changes observed on the protein level that would be compatible with eIF4F inhibition. We used next-generation RNA sequencing (RNA-seq) to perform unbiased analyses of transcriptome changes in melanoma cell lines in response to 20 h treatments with 100 nM RocA. The results of the differential expression analysis for A375 and MelJuso cells are presented in *SI Appendix*, Tables S3 and S4, respectively. Fig. 4 presents the 20 most significantly differentially expressed genes in RocA-treated A375 (Fig. 4 *A* and *C*) and MelJuso (Fig. 4 *B* and *D*) cells. Among transcripts most prominently up-regulated in both melanoma genetic contexts in response to eIF4Fi were mRNAs for transcription factors KLF6, EGR1, FOSL2, and IER2. Our quantitative RNA-seq analyses also revealed a significant upregulation of *DUSP5* and *DUSP6* expression in response to RocA in both melanoma genetic contexts (*SI Appendix*, Fig. S6 and Datasets S3 and S4), confirming that the suppression of DUSP6 expression in response to eIF4Fi is posttranscriptional.

We also performed an Ingenuity Pathway Analysis (IPA, Dataset S5) of the RNA-seq data to predict upstream regulators responsible for the observed transcriptome changes and perturbances in canonical pathways activity. Reassuringly, both analyses confirmed high degree of similarity in melanoma cell lines responses to eIF4Fi. Moreover, IPA also identified KLF6 and EGR1 among potential upstream regulators contributing to the changes in the expression profiles determined by RNA-seq. For further analyses, we selected the *EGR1* expression as a potential marker of ERK hyperactivation in melanoma, as the *EGR1* gene encodes a transcription factor known to act downstream of the ERK signaling pathway. Moreover, Gudernova et al. recently developed a luciferase reporter system based on the *EGR1* promoter that could be used to study changes in the RAS/RAF/MEK/ERK MAP kinase signaling quantitatively (29).

### ERK Hyperactivation Drives EGR1 Overexpression in Melanoma Cells.

**Fig. 5.**
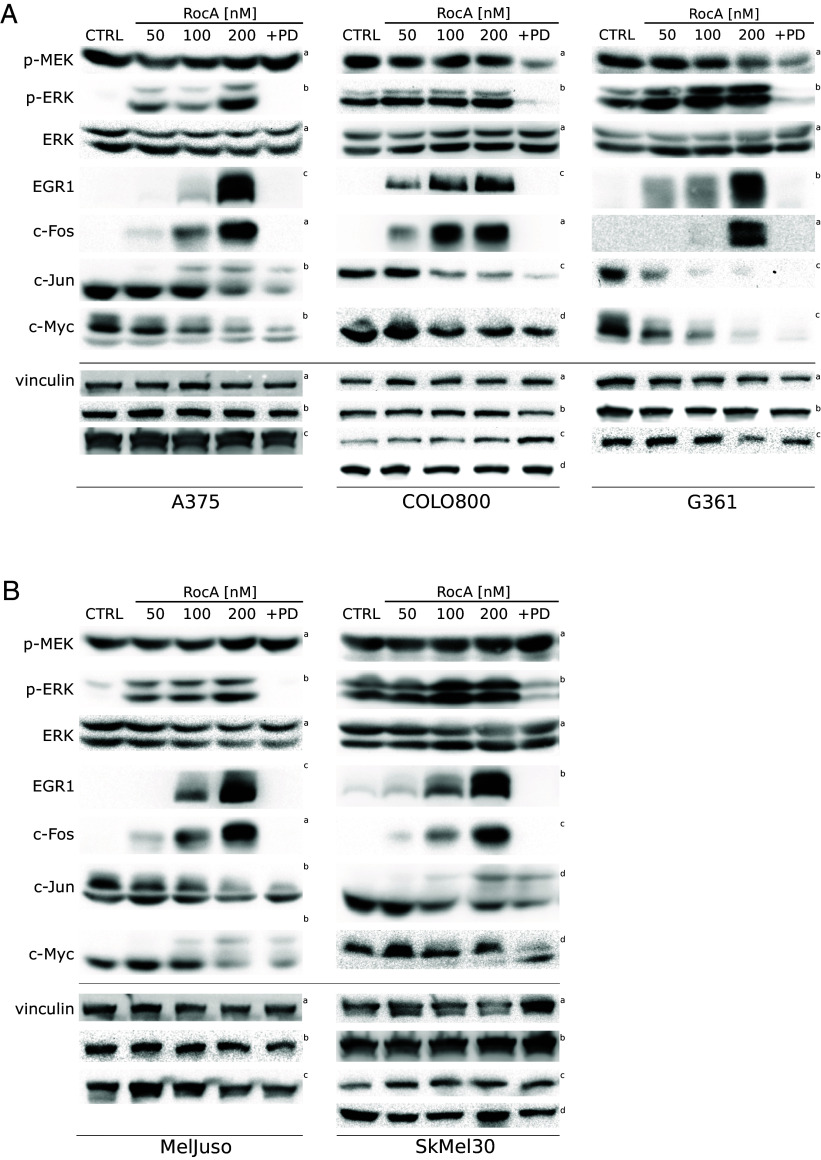
In a panel of melanoma cell lines, eIF4F inhibition promotes the hyperactivation of the ERK pathway, along with ERK-driven upregulation of EGR1 and c-Fos transcription factors. ERK activity and the levels of selected ERK downstream targets were analyzed by Western blot in (*A*) BRAF^V600E^-mutant (A375, COLO800, G361) and (*B*) *NRAS*-mutant (MelJuso, SKMel30) melanoma cells in response to a 20 h treatment with Rocaglamide A (RocA) at indicated concentrations. A combination of RocA and PD184352, a small-molecule MEK inhibitor (PD, 0.5 µM), confirmed that EGR1 and c-Fos upregulation is caused selectively by ERK hyperactivation. Vinculin served as a loading control. The control samples (CTRL) were treated with an equivalent volume of the vehicle (DMSO). The upper index refers to the corresponding loading control detected on the same membrane.

We performed Western blot analyses to determine changes in the levels of EGR1 in a panel of three BRAF^V600E^ melanoma cell lines (A375, COLO800, and G361). Importantly, all three cell lines responded to a 20 h treatment with RocA by ERK hyperactivation that could be completely abolished by the addition of a specific small-molecule MEK inhibitor PD184352 (Fig. 5*A*). These data supported the notion that the upstream oncogenic signaling was strongly amplified on the ERK level in eIF4F inhibitor-treated BRAF^V600E^ melanoma cells. For comparison, we also analyzed the levels of c-Myc, synthesized in an eIF4F-dependent manner due to its mRNA’s structured 5′ UTR (*SI Appendix*, Fig. S4*C*), requiring the eIF4F activity for translation (30, 31), and two other transcription factors serving as ERK downstream targets: c-Jun and c-Fos. While c-Myc and c-Jun expression proved sensitive to eIF4Fi and their protein levels were downregulated in RocA-treated BRAF^V600E^ melanoma cells, the protein levels of c-Fos and EGR1 positively correlated with increased ERK activity. Critically, the eIF4Fi-induced increase in EGR1 and c-Fos protein levels was entirely abrogated by the specific MEK inhibitor PD184352, confirming the complete dependence of the induced c-Fos and EGR1 overexpression in BRAF^V600E^ melanoma cells on the ERK pathway activity. Notably, essentially the same response to eIF4Fi was observed in *NRAS*-mutant lines (MelJuso, SkMel30; Fig. 5*B*), indicating that the strong upregulation of c-Fos and EGR1 transcription factors could be a universal response of human melanoma cells to eIF4Fi, potentially also contributing to their survival under the conditions of inhibited eIF4F activity.

Of note, unlike c-Myc and c-Jun, the EGR1 and c-Fos protein expression was low in untreated melanoma cells but was significantly induced in response to eiF4Fi, indicating that the increased EGR1 and c-Fos levels might be a sign of a disrupted control of the MAPK optimum or a shift of the MAPK optimum toward higher ERK activity levels. The observed changes in selected ERK targets’ gene expression in response to eIF4Fi seem to reflect the length of 5′ UTR sequences of their mRNA transcripts and their predicted structural complexity (*SI Appendix*, Fig. S4).

### *EGR1* and *FOS* Gene Expression Patterns Indicate Differences in ERK Signaling Intensity in Melanoma Tumor Samples.

To find out whether the variability in EGR1 and c-Fos expression is also present in tumor samples of melanoma patients, we analyzed data generated by the TheCancer Genome Atlas (TCGA) Research Network (https://www.cancer.gov/tcga) (32) using the UCSC Xena Functional Genomics Explorer (https://xenabrowser.net). A comparison of *EGR1*, *FOS, IER2, JUN, KLF6, MYC, and FOSL2* gene expression in 481 tumor samples revealed that individual tumors vary substantially in the expression of all analyzed genes (*SI Appendix*, Fig. S7*A*). Importantly, tumors highly expressing *EGR1* commonly also highly expressed *FOS and IER2*. Similarly, low *EGR1* expression often correlated with low *FOS* and low *IER2* expression. There did not seem to be a clear association between the *EGR1*, *FOS*, and *IER2* expression levels and the *BRAF* and *NRAS* mutational status. The *KLF6*, *MYC*, and *FOSL2* genes did not seem to follow the *EGR1/FOS* expression pattern in the analyzed tumor samples (*SI Appendix*, Fig. S7*A*). However, an *EGR1* -like pattern of gene expression was observed for *JUN*, indicating that the intensity of expression of the *EGR1*, *FOS*, *IER2*, and *JUN* transcription factor genes could be coregulated at least in some human melanomas (*SI Appendix*, Fig. S7*A*).

Our RNA-seq analyses showed significant upregulation of not only *EGR1* but also *DUSP5* and *DUSP6* expression in response to eIF4Fi in both melanoma genetic contexts (Datasets S3 and S4). In thyroid cancer, increased DUSP5 and DUSP6 MAPK phosphatase expression was associated with higher ERK activity in *BRAF-*mutant tumors (33). In human melanoma tumors, the *DUSP5* and *DUSP6* expression follows a pattern similar to that of *EGR1* and *FOS* (*SI Appendix*, Fig. S7*B*), suggesting that the expression of these genes at least partly reflects ERK pathway activity, which seems to be present at varying levels in individual patients, irrespective of the driving mutation.

### eIF4F Inhibition Unmasks a Significant Spare Capacity of Oncogenic ERK Signaling in Melanoma.

**Fig. 6.**
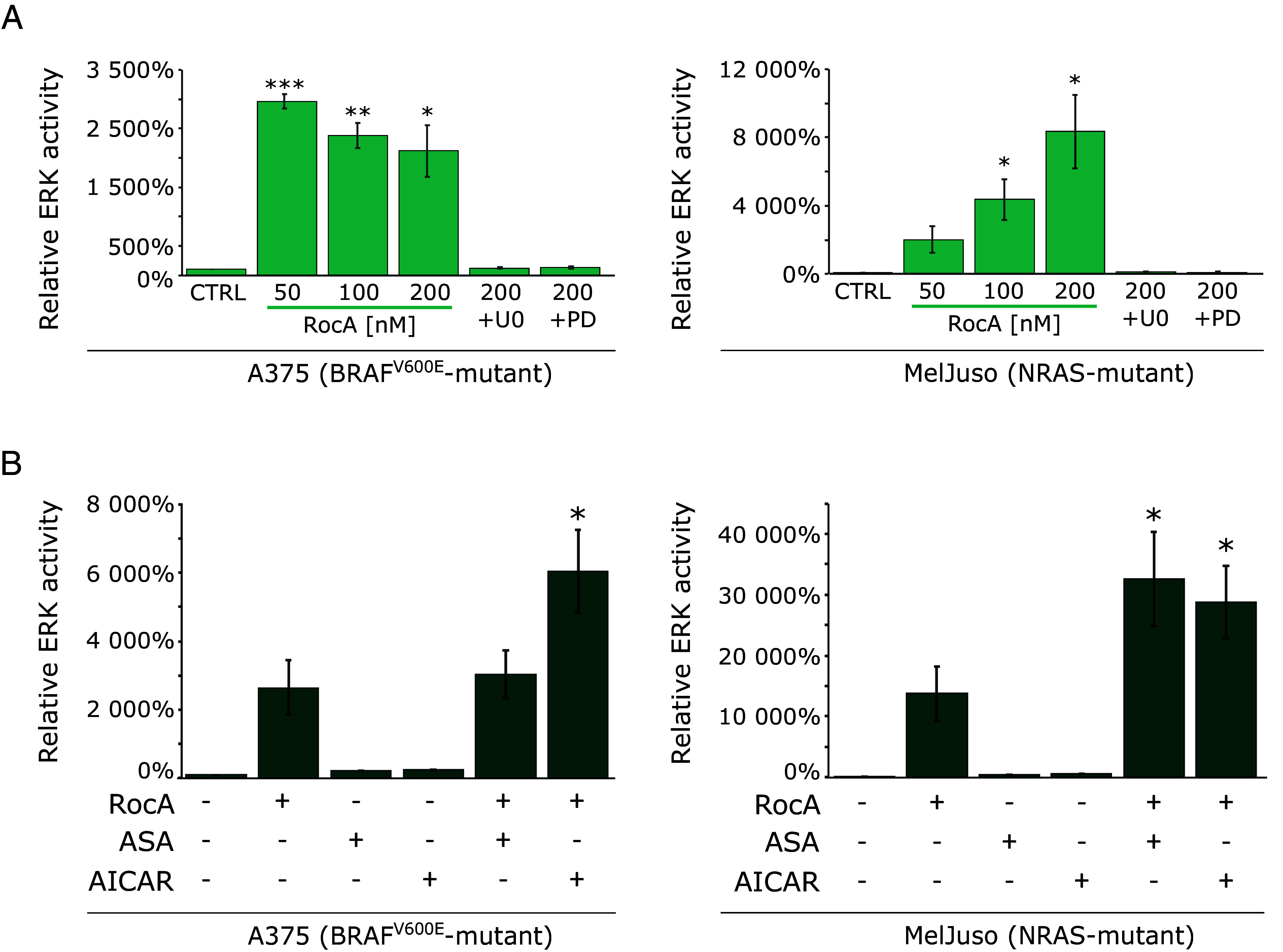
ERK pathway hyperactivation caused by eIF4F inhibition is amplified under metabolic stress conditions. (*A*) Quantitative analysis of the relative ERK activity in A375 and MelJuso cells increased in response to eIF4F inhibition. Cells, stably transfected with the reporter construct pKROX24(MapErk)Luc, expressing firefly luciferase under the control of an *EGR1* gene-derived promoter, were treated with Rocaglamide A alone or in combination with MEK inhibitors, U0126 (U0, 10 µM) or PD184352 (PD, 0.5 µM) for 24 h. (*B*) eIF4F inhibition and AMPK activation synergize in increasing the signaling capacity of the ERK pathway. A375 and MelJuso cells stably transfected with pKROX24(MapErk)Luc were treated with Rocaglamide A (200 nM) alone or in combination with AMPK activators Acetylsalicylic Acid (ASA, 5 mM) and 5-Aminoimidazole-4-carboxamide 1-β-D-ribofuranoside (AICAR, 2 mM) for 24 h. The control samples (CTRL) were treated with an equivalent volume of the vehicle (DMSO). Results were obtained from three independent repetitions and normalized to protein concentration. The data are presented by an average of ERK activity relative to the control samples ± SE. *P < 0.05.

Based on the above results, we decided to create ERK activity reporter cell lines by stably transfecting A375 and MelJuso cells with the reporter construct pKROX24(MapErk)Luc, expressing firefly luciferase under the control of an *EGR1* gene-derived promoter to study the eIF4F-mediated control of MAPK optimum in melanoma (29). Both melanoma genetic subtypes responded to 20 h treatments with 50, 100, and 200 nM RocA with a potent increase in the luciferase expression (Fig. 6*A*). In the MelJuso-derived reporter cell line, eIF4Fi induced an extremely high dose-dependent increase in luciferase activity, up to 80-fold compared to the DMSO-treated control, despite the presence of the oncogenic *NRAS* mutation already potently activating the ERK signaling pathway in MelJuso cells. ERK hyperactivation (>20-fold) was also observed in the A375-derived reporter cells bearing the BRAF^V600E^ activating mutation.

The results strongly supported the notion that eIF4F negatively controls ERK signaling and eIF4Fi-induced ERK hyperactivation stimulates the *EGR1* promoter activity in both *BRAF*- and *NRAS*-mutated melanoma cells. Critically, the eIF4Fi-induced upregulation of luciferase expression was completely inhibited by the addition of two small-molecule MEK inhibitors U0126 and PD184352, confirming the high specificity of the reporter system in melanoma cells. Moreover, this result again suggested that the strong ERK pathway signal originated from the mutant *NRAS* and *BRAF* oncogenes, passed through MEK, and was amplified, rather than generated, at the ERK level in response to eIF4Fi.

Even if we consider the fact that luciferase accumulated in the reporter cell lines over a more extended period, the robust upregulation of ERK-dependent transcription indicated that the ERK pathway must be strongly suppressed by the eIF4F/MKPs-mediated negative control mechanism to achieve the MAPK signaling optimum in melanoma cells. DUSPs rapidly inactivate ERK molecules activated by the upstream oncogenic signaling to maintain the ERK activity levels compatible with melanoma cell growth and proliferation.

In a previous study, we reported that AMPK activation by metabolic stress was able to enhance the oncogene-driven ERK signaling in melanoma cells upstream of MEK by promoting RAF–KSR interactions (34). Here, we used these findings to determine whether the extreme ERK signaling flux observed in eIF4Fi-treated cells already reached the maximal signaling capacity of melanoma cells. We combined RocA with two AMPK activators [acetylsalicylic acid (ASA) and AICAR], which might further stimulate the ERK pathway at the RAF kinase level. Compared to the effect of eIF4Fi, the AMPK activators on their own had only a minor impact on the ERK-dependent transcription (Fig. 6*B*). However, both AMPK activators synergistically potentiated RocA-induced ERK-dependent luciferase production in *NRAS*-mutated MelJuso melanoma cells, where the maximal luciferase activity was approximately 300-fold higher than the activity observed in untreated controls. In A375 cells, the addition of acetylsalicylic acid to RocA treatments did not seem to promote ERK-dependent transcription, while AICAR significantly enhanced eIF4Fi-induced luciferase production also in this genetic subtype. These results again support the notion that the MAPK optimum established in malignant melanoma cells bearing oncogenic *BRAF* and *NRAS* mutations is extremely low compared to the total signaling capacity of the ERK pathway in these cells. Our findings suggest that there is a wide window in human melanomas between the typical ERK signaling flux set by the negative feedback mechanisms and the oncogene-driven maximal ERK pathway capacity that could be exploited therapeutically. It should be noted that AMPK has also been reported to modulate eIF4F via the AMPK–MNK–eIF4E axis (35) and by inhibiting the mTORC1 complex, leading to eIF4E binding to its negative regulator 4EBP1 (36, 37). However, we did not expect these mechanisms to significantly impact eIF4F activity in RocA-treated cells. As already mentioned, rocaglates inhibit and sequester eIF4A, and the inhibition of translation initiation by rocaglates was proved to be independent of MNK and eIF4E phosphorylation (16, 17, 24).

### eIF4F Inhibition Promotes ERK Hyperactivation in Melanoma Tumors In Vivo.

**Fig. 7.**
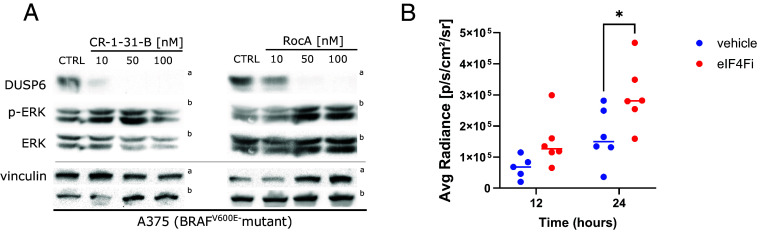
eIF4F inhibition promotes ERK hyperactivation in melanoma tumors in vivo. (*A*) The synthetic rocaglate CR-1-31-B downregulates DUSP6 and promotes ERK hyperactivation in A375 melanoma in vitro. Cells were treated with the indicated concentrations of CR-1-31-B and RocA for 20 h, and cell lysates were analyzed using Western blotting. The control samples (CTRL) were treated with an equivalent volume of the vehicle (DMSO). The upper index letters refer to the corresponding loading control detected on the same membrane. (*B*) ERK activity in orthotopic melanoma xenografts [A375 cells stably transfected with pKROX24(MapErk)Luc] was noninvasively monitored 12 and 24 h posttreatment with eIF4Fi (CR-1-31-B, 0.2 mg per kg in sesame oil, i.p.) using a bioluminescent signal (n = 5 to 6 per time point). The data are presented as average radiance [p/s/cm^2^/sr] (each dot represents an individual animal, line ~ median). *P < 0.05 (One-Way ANOVA, Tukey’s contrast).

Next, we performed experiments to determine whether eIF4Fi could elicit ERK hyperactivation in tumors in vivo. We decided to use CR-1-31-B, a synthetic rocaglate with more favorable pharmacokinetic properties than RocA, which has already demonstrated antitumor activity in mouse models, e.g., in KRAS-driven pancreatic cancer (38). First, we analyzed the impact of CR-1-31-B on DUSP6 levels and the ERK pathway activity in A375 melanoma cells using Western blot (Fig. 7*A*). As CR-1-31-B recapitulated the RocA activities, we proceeded to the in vivo experiment. Mice were injected with A375 cells stably transfected with the ERK activity reporter construct pKROX24(MapErk)Luc. When tumors formed, mice were treated with 0.2 mg/kg CR-1-31-B, and ERK activity in tumor cells was determined by in vivo imaging 12 and 24 h later (Fig. 7*B*). A trend toward increased luciferase activity in tumors was observed already after 12 h treatment with CR-1-31-B. After 24 h, the difference in average radiance between vehicle-treated and eIF4Fi-treated animals was statistically significant. These data indicate that pharmacological eIF4F inhibition could promote ERK pathway hyperactivation in *BRAF*-driven melanoma tumors in vivo.

## Discussion

The treatment of metastatic melanoma has been challenging because of its intrinsic resistance to standard chemotherapy and radiotherapy. Identification of the essential role of the oncogenic *BRAF* and *NRAS* mutations activating ERK signaling for melanoma development and survival has led to the development of pharmacological BRAF and MEK inhibitors. They are highly effective treatments, especially when combined, for melanomas bearing the most common BRAF^V600E^ mutation, prolonging the survival of melanoma patients (2, 39). However, resistance develops in most patients, leading to relapse and progression.

The eIF4F translation initiation complex has been identified as an essential player in the development of melanoma resistance to clinical drugs targeting BRAF and MEK kinases (10, 11, 13). In this study, we provide insights into the role of the eIF4F translation initiation complex in the negative regulation of the RAS/RAF/MEK/ERK MAPK signaling pathway in melanoma cells. We demonstrate that eIF4F is essential for maintaining optimal ERK signaling intensity in treatment-naïve melanoma cells harboring BRAF or NRAS mutations. The concept of a tightly controlled ERK MAPK fitness zone could explain the observation that melanoma cells with RAF and RAS mutations tolerate neither too low nor excessively high ERK pathway activity (6–8). Our findings not only shed light on the underlying mechanisms of eIF4F-mediated resistance to BRAF and MEK inhibitors in melanoma, but they also indicate the possibility of pharmacologically disrupting critical control mechanisms adjusting active ERK levels and allowing melanoma cells to thrive despite potent activating mutations in the ERK pathway. Interestingly, similar to the ERK MAPK fitness zone, there also seems to be a cancer cell fitness zone associated with the levels of eIF4F activity (40).

One of the key findings of our study is the identification of DUSP6/MKP3 as a downstream effector of eIF4F in the negative feedback regulation of the ERK pathway in melanoma. DUSP6 acts as a phosphatase that deactivates ERK, providing a crucial negative feedback loop to prevent excessive ERK activation (26). While in normal cells, this feedback also limits the duration of the response to stimuli, in cells bearing mutations constitutively activating the signaling, the negative feedback helps maintain optimal ERK pathway intensity compatible with tumor cell survival and growth (4, 41). The concept of ERK activity fitness zone and its feedback control by DUSP6 might help to explain somewhat conflicting observations concerning the role of this phosphatase in cancer, where in some cellular contexts, DUSP6 behaves as an oncogene while in others, it seems to act as a tumor suppressor (25). We show that eIF4F is required for the continuous production of DUSP6, which limits aberrant ERK signaling driven by oncogenic *BRAF* and *NRAS*. Inhibition of eIF4F disrupts this negative feedback control and leads to hyperactivation of ERK. This suggests that eIF4F plays a critical role in maintaining the balance of ERK signaling in melanoma cells. Importantly, our findings might be relevant to other types of cancer. A recent study showed that ERK hyperactivation is toxic to lung adenocarcinoma driven by RTK and *KRAS* mutations. The same eIF4F-dependent control mechanism could be at play to restrain the oncogene-driven ERK signaling, as DUSP6 was also implicated in limiting ERK activity in this context (42). Moreover, DUSP6 levels and ERK activity were recently reported to be under translational control in *KRAS*-mutant lung adenocarcinomas (43).

Our results may also provide insight into the mechanisms of ERK pathway activation in *NRAS*-mutant melanomas. The signal from mutated NRAS protein is believed to be transmitted predominantly via the CRAF kinase (44). Interestingly, the eIF4F inhibitor Rocaglamide A used in our study can also disrupt an essential functional interaction between CRAF and prohibitin (PHB) proteins that promotes CRAF activation (45). The very same molecular mechanism was implicated in the negative effect of Rocaglamide A on ERK pathway activity in *KRAS*-driven lung cancer (46), although the picture might be somewhat complicated by the finding that *KRAS* oncogene expression could be eIF4F-dependent, at least in some tumor types (47). In any case, we expected ERK signaling in melanoma cells bearing *NRAS* mutations to be highly sensitive to RocA. While the observed decrease in active MEK levels in RocA-treated *NRAS*-mutant melanoma cells suggests that CRAF–PHB interactions may partly contribute to the control of ERK signaling, the concomitant loss of the negative feedback control leads to potent ERK hyperactivation also in this cellular context.

Our study also highlights the extent of spare signaling capacity in the ERK pathway in melanoma cells with *BRAF* or *NRAS* mutations. We find that under normal conditions, the majority of ERK molecules in melanoma cells are kept inactive through the eIF4F-dependent feedback mechanisms. Whether such extensive spare capacity might be fully utilized under physiological conditions to allow for a rapid response of melanoma cells to specific signals remains to be investigated. The ERK MAPK fitness zone in melanoma remains largely uncharacterized. It is not known how much of the full signaling capacity of the ERK pathway is used to stimulate cancer growth and by which extent the intensity of the ERK signaling driven by oncogenic mutations has to be suppressed by various feedbacks to allow for cancer cell proliferation. It is also not clear, what the minimal activity of the pathway is that allows melanoma growth and what the maximum activity is beyond which the cells cease to proliferate or lose viability. Small-molecule eIF4F inhibitors could serve as tools for studying the maximal signaling capacity of the ERK pathway naturally present in tumor cells.

Furthermore, our results indicate that the intensity of ERK signaling might differentially affect various ERK targets. Some targets might only be strongly expressed when ERK activity reaches a higher threshold. Treatment with eIF4F inhibitors leading to ERK hyperactivation promoted overexpression of EGR1 and c-Fos, whose expression was relatively low in normally growing melanoma cells. It is worth noting that while eIF4F inhibition immediately impairs protein synthesis, melanoma cells can remain viable for several days. This suggests that short-lived proteins are not essential for viability and/or the existence of compensatory mechanisms that sustain cell survival in the context of reduced protein synthesis. Further investigations are warranted to elucidate the roles of EGR1 and c-Fos in these potential compensatory mechanisms and identify vulnerabilities that could be targeted in combination with eIF4F inhibitors. Interestingly, c-Fos was also identified as a candidate MAPK pathway inhibitor resistance gene in near genome-scale ORF/cDNA screens and its overexpression promoted melanoma cell resistance to RAF, MEK, and ERK inhibitors (48).

**Fig. 8.**
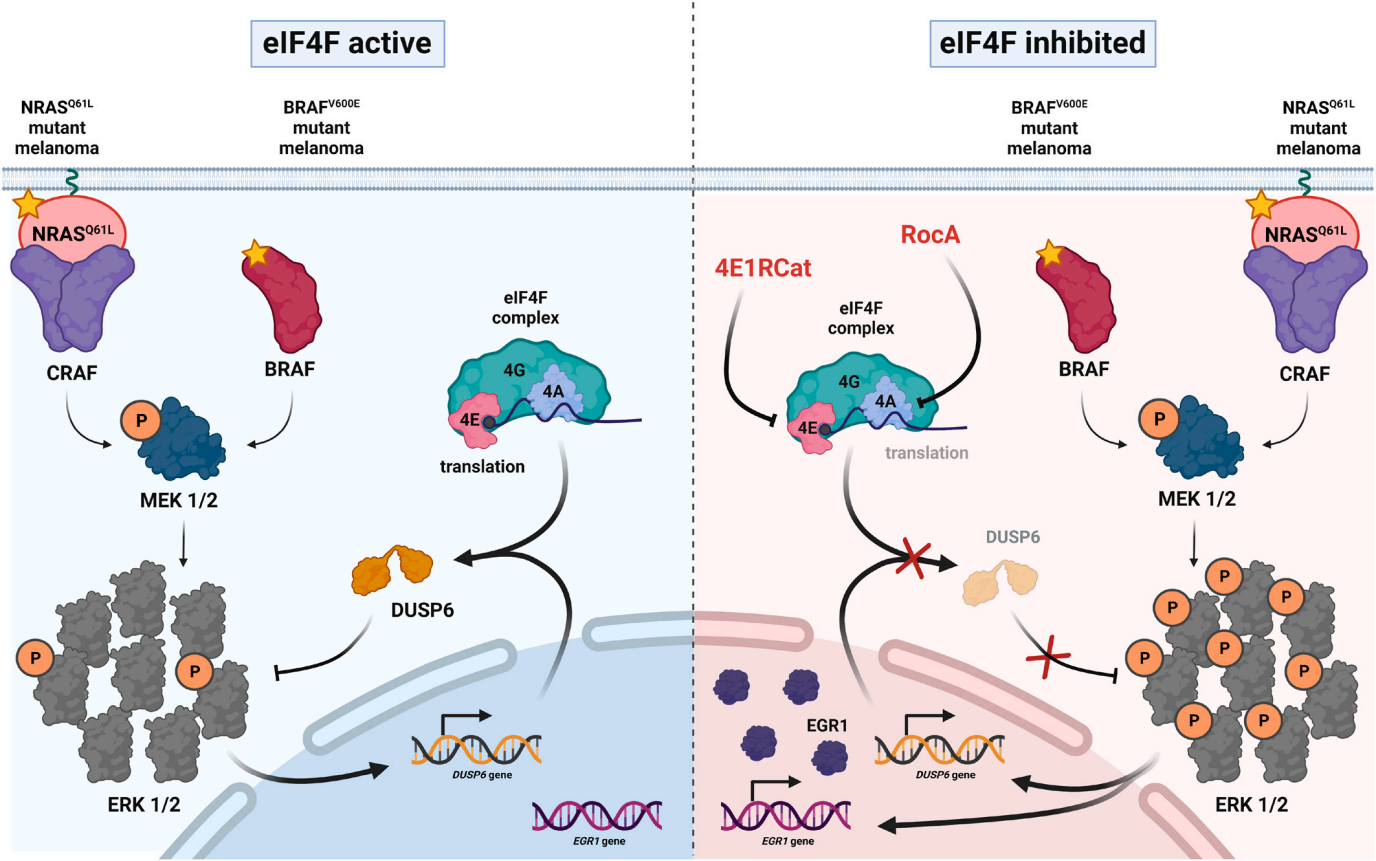
eIF4F activity controls ERK signaling intensity in melanoma. A model describing the role of eIF4F in maintaining optimal ERK pathway signaling flux and the mechanism of ERK hyperactivation in melanoma cells bearing BRAF and NRAS oncogenic mutations in response to small-molecule eIF4F inhibitors. Created with BioRender.com.

In conclusion, our study reveals a critical role for eIF4F in the negative regulation of the ERK MAPK pathway in melanoma. By controlling the production of DUSP6 and maintaining spare signaling capacity, eIF4F contributes to the optimal ERK signaling flux in treatment-naïve melanoma cells. Pharmacological inhibition of eIF4F disrupts this balance and promotes hyperactivation of the ERK pathway (Fig. 8). These findings provide a rationale for targeting eIF4F as a potential therapeutic strategy not only to overcome resistance to BRAF and MEK inhibitors but also for treatment-naïve melanomas. Further studies are needed to precisely characterize the MAPK optimum, explore the therapeutic potential of eIF4F inhibitors, and unravel the compensatory mechanisms that sustain melanoma cell survival in the context of reduced protein synthesis. Overall, our findings contribute to a better understanding of the molecular mechanisms underlying melanoma resistance to targeted therapies and provide insights into potential strategies to improve treatment outcomes for patients with *BRAF-* or *NRAS*-mutant melanomas.

## Materials and Methods

### Cell Culture and Treatments.

Human cell lines A375, COLO800, G361, MelJuso, and SkMel30 were obtained from the European Collection of Animal Cell Cultures (ECACC) and the German Collection of Microorganisms and Cell Cultures (DSMZ). The cell lines were cultured in RPMI 1640 medium (Sigma-Aldrich) supplemented with 10% fetal bovine serum (Sigma-Aldrich), 1% penicillin/streptomycin, and 2 mM L-glutamine (both Gibco), and regularly *Mycoplasma*-tested. Rocaglamide A, 4E1RCat, CR-1-31-B, and AICAR were purchased from MedChemExpress, U0126 from Wako Chemicals, MG132 from Cayman Chemical, acetylsalicylic acid from Sigma-Aldrich, and PD184352 from Selleckchem. AICAR was dissolved directly in the culture media. Stock solutions of the other compounds were prepared in dimethyl sulfoxide (DMSO), and control samples were treated with the corresponding amount of the vehicle.

### Transfection and Luciferase Assay.

The A375 and MelJuso cell lines were stably transfected with the ERK activity luciferase reporter construct pKROX24(MapErk)Luc (available from Addgene, #200112) using TurboFect Transfection Reagent (Thermo Fisher Scientific), following the manufacturer’s instructions. Hygromycin was added to the growth media (100 μg/mL) 24 h posttransfection, and pools of stable transfectants were selected for 10 d. Luciferase Assay System Kit (Promega) was used according to the manufacturer's recommendations to determine the ERK activity. Cells were lysed on ice and the luminescence was measured in the TriStar^2^ LB 942 reader (Berthold Technologies). To calculate relative ERK activity, the luminescence values were normalized to total protein concentrations determined using the Bio-Rad Protein Assay Dye Reagent (#5000006). Cells were transfected using the Lipofectamine™ RNAiMAX Transfection Reagent (Thermo Fisher Scientific) for siRNA-mediated knockdown of gene expression, following the manufacturer’s instructions. The gene-specific siRNAs were purchased from Santa Cruz Biotechnology: eIF4E siRNA (h) (sc-35284), eIF4G siRNA (h) (sc-35286), and eIF4AI siRNA (h) (sc-40554). AllStars Negative Control siRNAs were purchased from QIAGEN.

### Western Blot.

Whole cell lysates were prepared using 2× Laemmli Sample Buffer, resolved on SDS-PAGE gels, and transferred to PVDF membranes (Merck Millipore). The membranes were blocked in 5% fat-free milk in Tris-buffered saline containing 0.1% Tween-20 (TBST) for 1 h at room temperature and incubated with primary antibodies at 4 °C overnight. Then the membranes were washed 4 × 7 min with TBST and incubated with horseradish peroxidase-linked secondary antibodies for 1 h at room temperature. After washing for 4 × 7 min, the membranes were exposed to enhanced luminescence (ECL) substrate and signal detected in the G-Box detection system (Syngene). For sequential detection, membranes were stripped using Restore™ Western Blot Stripping Buffer (Thermo Fisher Scientific).

### Statistical Analysis.

Each luciferase assay was performed with at least three biological repetitions comparing controls with experimental conditions. Results are presented as mean ± SEM. Statistical significance was evaluated using Student’s two-tailed *t* test, and *P*-values *P* < 0.05 were considered statistically significant.

## Supplementary Material

Appendix 01 (PDF)

Dataset S01 (XLSX)

Dataset S02 (XLSX)

Dataset S03 (XLSX)

Dataset S04 (XLSX)

Dataset S05 (XLSX)

## Data Availability

The RNA-seq datasets have been deposited at NCBI Gene Expression Omnibus under accession number GSE273721 (49). Additional information on antibodies, cell staining, the identification of MAPK targets by mass spectrometry-based proteomics, the puromycylation assay, RNA-seq analyses, and the in vivo experiment is provided in *SI Appendix*, *Materials and Methods*. Previously published data were used for this work. TCGA Program, https://xenabrowser.net. All other data are included in the manuscript and/or supporting information.
