# Supplementary material for: eIF4F controls ERK MAPK signaling in melanomas with BRAF and NRAS mutations
Source: Proc Natl Acad Sci U S A. 2024 Oct 22;121(44):e2321305121. doi: 10.1073/pnas.2321305121 (PMC11536119; doi:10.1073/pnas.2321305121)
Supplement: Supplementary file 1 — Appendix 01 (PDF) [file pnas.2321305121.sapp.pdf]

Supporting Information for

eIF4F controls ERK MAPK signaling in melanomas with *BRAF* and *NRAS* mutations

Content:

SI Materials and Methods	page 2
Figure S1	page 5
Figure S2	page 6
Figure S3	page 8
Figure S4	page 9
Figure S5	page 17
Figure S6	page 18
Figure S7	page 19
SI datasets S1-S5 (provided as separate MS Excel files)	page 21

SI Materials and Methods

Puromycylation assay. To assess the rate of protein synthesis, cell cultures were labeled for 15 minutes with 91 µM puromycin (Cayman). To visualize nascent polypeptides, cells were lysed for western blot analysis, and a monoclonal anti-Puromycin antibody, clone 12D10 (Merck Millipore, #MABE343), was used for the immunoblot detection.

Cell staining. Cells were washed post-treatment twice with PBS, and a mixture of 6% glutaraldehyde and 0.5% crystal violet (Sigma-Aldrich) was added for 30 minutes. The dishes were then rinsed with tap water and left to dry at room temperature.

Identification of MAPK targets by mass spectrometry. In this analysis, MAPK target proteins were immunoprecipitated from lysates of control and RocA-treated melanoma cells using beads with antibodies recognizing phosphorylated MAPK consensus target motif, and individual proteins were identified and quantified using mass spectrometry.

The A375 and MelJuso cell lines were seeded in 10-mm dishes and, on the following day, treated with RocA. Controls were treated with DMSO. 20 h post-treatment, cells were washed in cold PBS and collected on ice by scraping into the lysis buffer (20 mM Tris-HCl pH 7.5, 150 mM NaCl, 1 % NP-40, 1 mM Na_2 EDTA, and 1 mM EGTA) supplemented with protease and phosphatase inhibitors (Roche). Subsequently, the samples were immunoprecipitated with PTMScan® Phospho-MAPK/CDK Substrate Motif (PXS*P and S*PXK/R) Kit (Cell Signaling Technology), eluted in elution buffer (2 M urea, 50 mM Tris-HCl pH 7.5), and supplemented with 1:50 sequencing grade modified trypsin (Promega) and 1 mM DTT. After the overnight digestion at room temperature, TFA was added to stop the digestion, and samples were transferred to C18 Stage Tips. The tryptic peptides eluted from StageTips (80% ACN, 0.1% TFA) were lyophilized and resuspended in 0.1% TFA. Samples were analyzed on a Fuson Lumos mass spectrometer connected to an Ultimate Ultra3000 chromatography system (Thermo Scientific) incorporating an autosampler. 5 µL of each tryptic peptide sample was loaded on an Aurora column (IonOptiks, 250 mm length) and separated by an increasing ACN gradient, using a 40 min reverse-phase gradient (from 3%–40% ACN) at a flow rate of 400 nL/min. The mass spectrometer was operated with a cycle time of 1 s at 240k OT resolution, switching to IT MS/MS operated in “rapid” mode with a fixed resolution of 145-1450Da and an accumulation time of max 50ms with a 100% target.

MaxQuant version 1.6. was used for mass spectra analysis and peptide identification by searching against the UniProt Human database with the standard, preset settings. The LFQ (label-free quantitation) intensities from MaxQuant proteinGroups.txt output were used, and contaminants and reverse proteins were removed from the list.

Antibodies. The following primary antibodies were used for the detection of proteins of interest: monoclonal anti-puromycin antibody, clone 12D10 (Merck Millipore, #MABE343), monoclonal rabbit anti-phospho-MEK1/2 (#9154), monoclonal rabbit anti-phospho-ERK1/2 (#4370), polyclonal rabbit anti-ERK1/2 (#9102), monoclonal rabbit anti-eIF4A (#2013), monoclonal rabbit anti-eIF4E (#2067), polyclonal rabbit phospho-eIF4E (Ser209) Antibody (#9741), polyclonal rabbit anti-eIF4G (#2498), monoclonal rabbit anti-EGR1 (#4153), monoclonal rabbit anti-c-Fos (#2250), monoclonal rabbit anti-c-Jun (#9165), monoclonal rabbit anti-c-Myc (#5605), and monoclonal rabbit anti-DUSP4 (#5149) from Cell Signaling Technologies, and monoclonal mouse anti-DUSP5 (sc-393801), monoclonal mouse anti-DUSP6 (sc-377070), monoclonal mouse anti-DUSP7 (sc-377106), monoclonal mouse anti-DUSP9 (sc-137010),

monoclonal mouse anti-DUSP12 (sc-390760), and monoclonal mouse anti-vinculin (sc-73614) from Santa Cruz Biotechnology. Anti-PCNA mouse monoclonal antibody (clone PC10) was kindly provided by Dr. Borivoj Vojtesek. Mouse anti-rabbit IgG-HRP (sc-2357) and mouse IgG kappa binding protein (m-IgGκ BP) conjugated to horseradish peroxidase (sc-516102), both from Santa Cruz Biotechnology, were used for the detection.

RNA-seq and data analysis. Melanoma cells were seeded in 12-well cell culture plates and treated with 100 nM Rocaglamide A or DMSO (vehicle) 24 hours later. Total RNA was isolated 20 h post-treatment using RNAeasy Mini Kit (QIAGEN). Cells were lysed directly in the plates. RNA-seq data were obtained using Lexogen Quantseq FWD kit for Illumina with polyA selection and sequenced on Illumina sequencer (run length 1x75 nt). Bcl files were converted to Fastq format using bcl2fastq v. 2.20.0.422 Illumina software for basecalling. Quality check of raw single-end fastq reads was carried out by FastQC (1). The adapters and quality trimming of raw fastq reads was performed using Trimmomatic v0.39 (2) with settings CROP:250 LEADING:3 TRAILING:3 SLIDINGWINDOW:4:5 MINLEN:35. Trimmed RNA-Seq reads were mapped against the mouse genome (mm38) and Ensembl GRCm38 v.93 annotation using STAR v2.7.3a (3) as splice-aware short read aligner and default parameters except --outFilterMismatchNoverLmax 0.66 and --twopassMode Basic. Quality control after alignment concerning the number and percentage of uniquely- and multi-mapped reads, rRNA contamination, mapped regions, read coverage distribution, strand specificity, gene biotypes and PCR duplication was performed using several tools namely RSeQC v4.0.0 (4), Picard toolkit v2.25.6 (5), Qualimap v.2.2.2 (6).

The differential gene expression analysis was calculated based on the gene counts produced using featureCounts from Subread package v2.0 (7) and further analyzed by Bioconductor package DESeq2 v1.34.0 (8). Data generated by DESeq2 with independent filtering were selected for the differential gene expression analysis due to its conservative features and to avoid potential false positive results. Genes were considered as differentially expressed based on a cut-off of adjusted p-value ≤ 0.05 and log2(fold-change) ≥1 or ≤-1. Clustered heatmaps were generated from selected top differentially regulated genes using R package pheatmap v1.0.12 (9) and volcano plots were produced using ggplot2 v3.3.5 package (10). Ingenuity Pathway Analysis of RNA-seq data was performed to predict the upstream regulators and changes in canonical pathways using QIAGEN IPA (Summer Release 2023) in the default setting. The cut-off criteria for up- and downregulated genes described above were also used in the joint IPA analysis.

***In vivo* experiment.** Male NOD.Cg-Rag1^{tm1Mom} Il2rg^{tm1Wjl}/SzJ (NRG, The Jackson Laboratory, Bar Harbor, ME) mice aged six to eight weeks were anesthetized with a solution of ketamine (9.5 mg/ml; 99/192/85-C, Bioveta, Ivanovice na Hané, Czech Republic) and xylazine (0.95 mg/ml; A6A066, Bioveta, Ivanovice na Hané, Czech Republic) at a final dose of 100 µl/10 g of body weight, administered intraperitoneally. The mice were injected intradermally with 5×10^4 A375 cells (stably transfected with the ERK activity luciferase reporter construct pKROX24(MapErk)Luc) in 50 µl PBS. After 19 days, mice were treated with the eIF4F inhibitor CR-1-31-B (0.2 mg per kg, in sesame oil, i.p.). Live cell imaging was performed 12 and 24 h later using the IVIS Lumina XR optical imaging system (Perkin Elmer, Waltham, MA) 15 minutes after intraperitoneal injection of D-luciferin, sodium salt (LUCNA-10G, Goldbio, St. Louis, MO, 150 mg/kg) with automatic exposure times. Luminescence (Avg Radiance [p/s/cm²/sr]) was quantified using Living Image v4.7.2 software (Perkin Elmer, Waltham, MA). All experiments were conducted with the approval of the Academy of Sciences of the Czech Republic (AVCR 55/2024), overseen by the local ethical committee, and performed by certified individuals (R.V., O.V., K.So.).

1. S. Andrews, *FastQC: A Quality Control Tool for High Throughput Sequence Data* (2010). http://www.bioinformatics.babraham.ac.uk/projects/fastqc/

2. A. M. Bolger, M. Lohse, B. Usadel, Trimmomatic: A flexible trimmer for Illumina sequence data. *Bioinformatics* **30**, 2114–2120 (2014).

3. A. Dobin, *et al.*, STAR: Ultrafast universal RNA-seq aligner. *Bioinformatics* **29**, 15–21 (2013).

4. L. Wang, S. Wang, W. Li, RSeQC: quality control of RNA-seq experiments. *Bioinformatics* **28**, 2184–2185 (2012).

5. Picard Tools. Broad Institute, GitHub Repository (2018). http://broadinstitute.github.io/picard/

6. K. Okonechnikov, A. Conesa, F. García-Alcalde, Qualimap 2: advanced multi-sample quality control for high-throughput sequencing data. *Bioinformatics* **32**, 292–294 (2016).

7. Y. Liao, G. K. Smyth, W. Shi, featureCounts: an efficient general purpose program for assigning sequence reads to genomic features. *Bioinformatics* **30**, 923–930 (2014).

8. M. I. Love, W. Huber, S. Anders, Moderated estimation of fold change and dispersion for RNA-seq data with DESeq2. *Genome Biol.* **15**, 550 (2014).

9. R. Kolde, M. R. Kolde, *Package "pheatmap"* (2015). https://cran.ms.unimelb.edu.au

10. H. Wickham, Ggplot2. *Wiley Interdiscip. Rev. Comput. Stat.* **3**, 180–185 (2011).

Figure S1

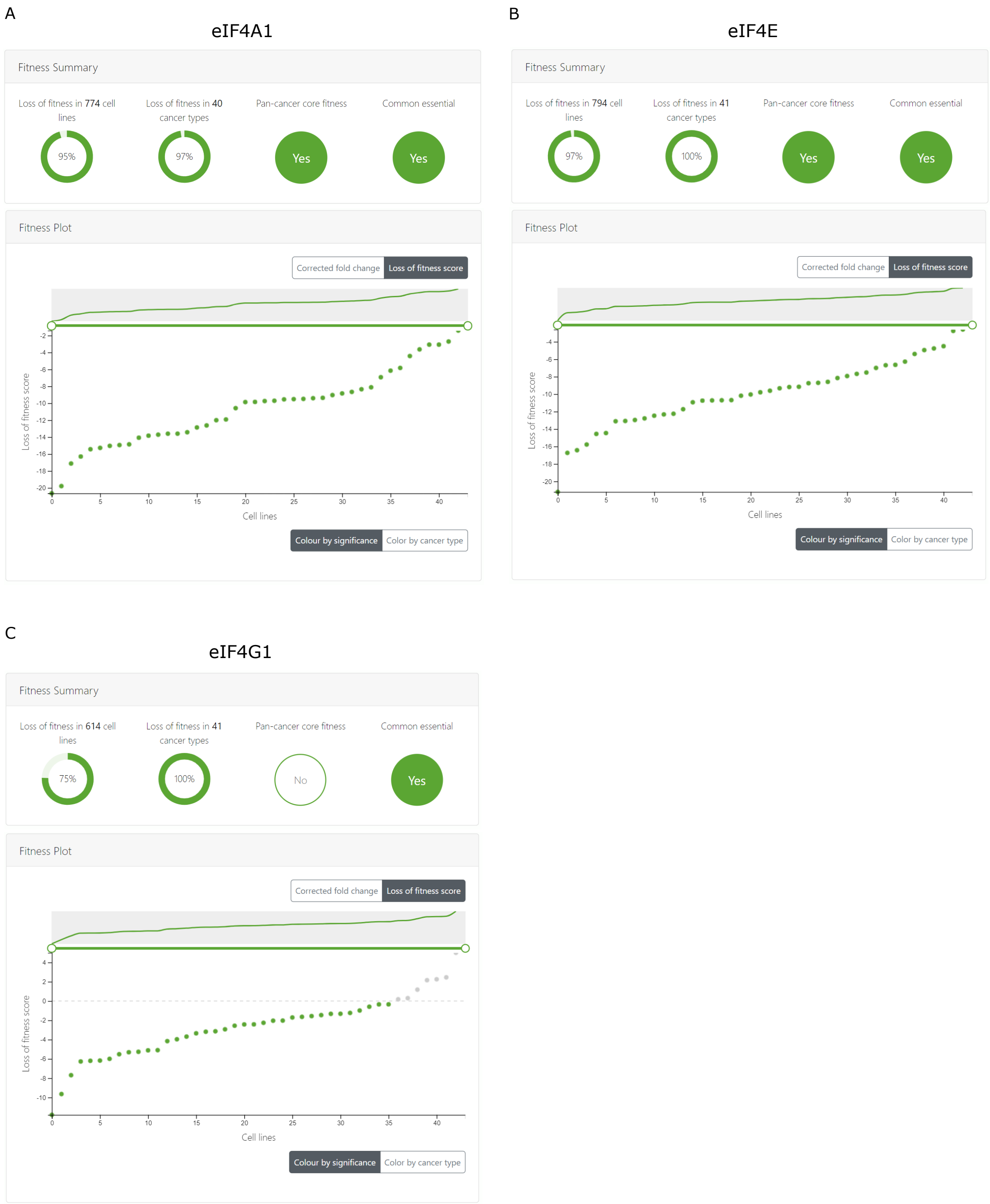

Figure S1: Knock-out of individual eIF4F complex subunits affects the fitness of cancer cells, including melanoma. The data were generated in Project Score (https://score.depmap.sanger.ac.uk), in which systematic genome-scale CRISPR-Cas9 drop-out screens in a large number of highly annotated cancer models were used to identify genes required for cell fitness in defined molecular contexts. Data are presented here for the eIF4F-encoding genes *eIF4A1* **(A)**, *eIF4E* **(B)**, and *eIF4G1* **(C)**. The top panel (Fitness Summary) summarizes the impact on multiple cancer cell types, and the bottom panel (Fitness Plot) shows the impact on the fitness of 43 human melanoma cell lines.

Figure S2

A

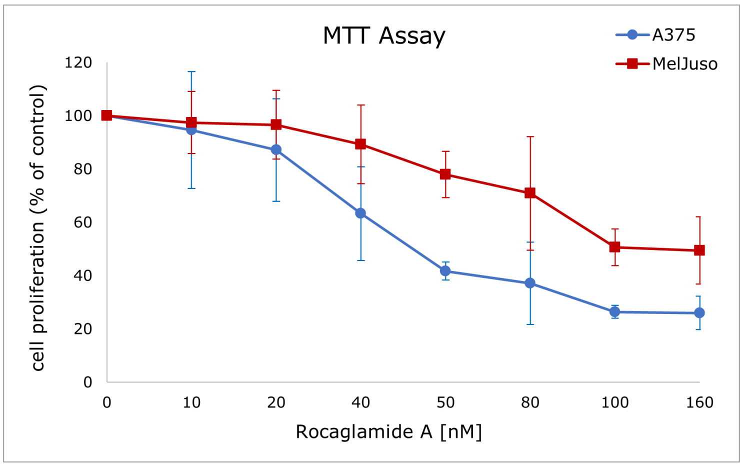

B

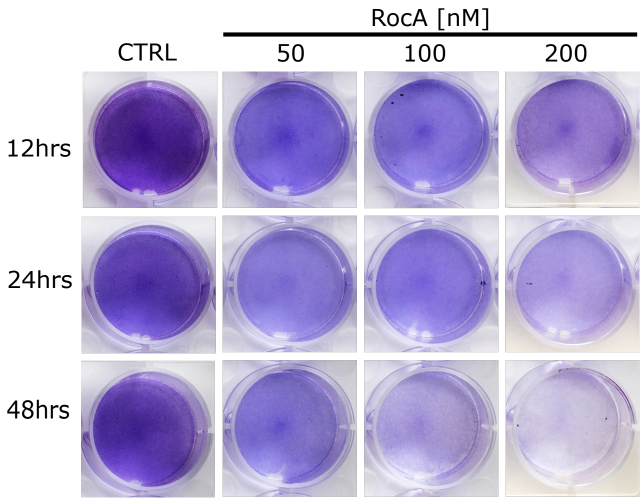

A375 (BRAF^{V600E}-mutant)

C

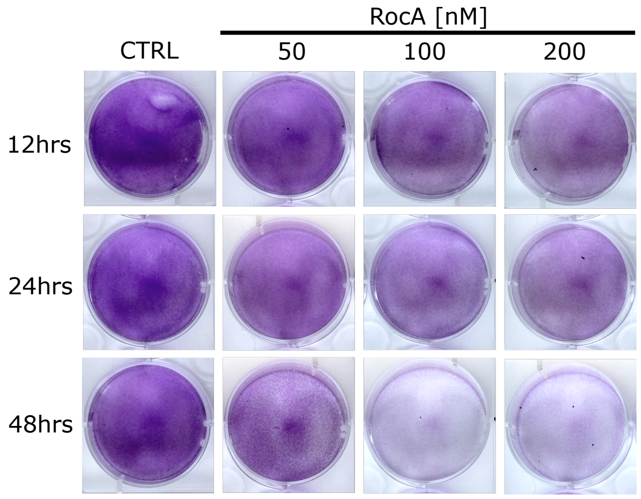

MelJuso (NRAS-mutant)

Figure S2: Impact of Rocaglamide A on melanoma cell proliferation and viability. (A) MTT assays were performed on A375 and MelJuso cells to determine cell viability and proliferation upon eIF4F inhibition. Cells were treated with the indicated concentrations of Rocaglamide A for 48 h, washed and incubated with 3-(4,5-dimethylthiazol-2-yl)-2,5-diphenyltetrazolium bromide (MTT, 0.5 mg/ml) for 4 h at 37°C. The graph represents three independent repetitions, and data are presented as the proportion of metabolically active cells relative to the control sample (mean) ± standard error of the mean, represented by error bars. The control samples (0 nM) were treated with the vehicle (DMSO) volume equivalent to the DMSO volume in the highest RocA concentration. Staining of A375 **(B)** and MelJuso **(C)** cell cultures treated with Rocaglamide A (RocA) for 12, 24, and 48 h. Cells were treated with the indicated RocA concentrations, washed, and fixed/stained with 6 % glutaraldehyde + 0.5 % crystal violet solution.

Figure S3

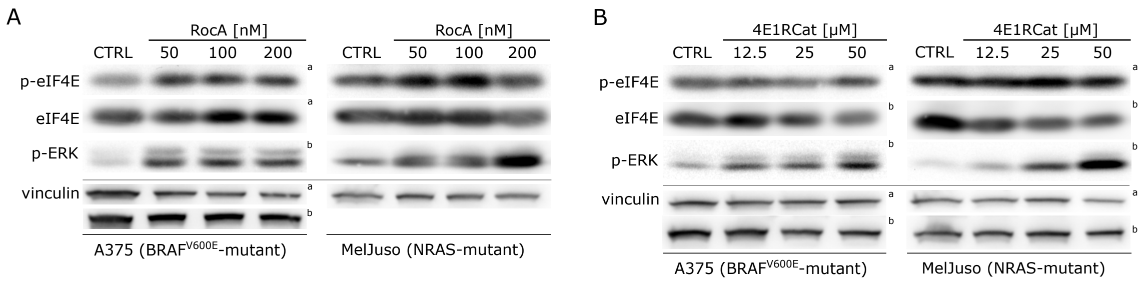

Figure S3: Impact of eIF4F inhibition on eIF4E phosphorylation levels in melanoma cells. Western blot analysis of A375 and MelJuso cells after 20 h treatment with increasing concentrations of small-molecule eIF4F inhibitors: **(A)** eIF4A inhibitor Rocaglamide A (RocA) or **(B)** eIF4E-eIF4G disruptor, 4E1RCat. Vinculin served as a loading control. The control samples (CTRL) were treated with an equivalent volume of the vehicle (DMSO). The upper index letters refer to the corresponding loading control detected on the same membrane.

Figure S4

A) *DUSP6*

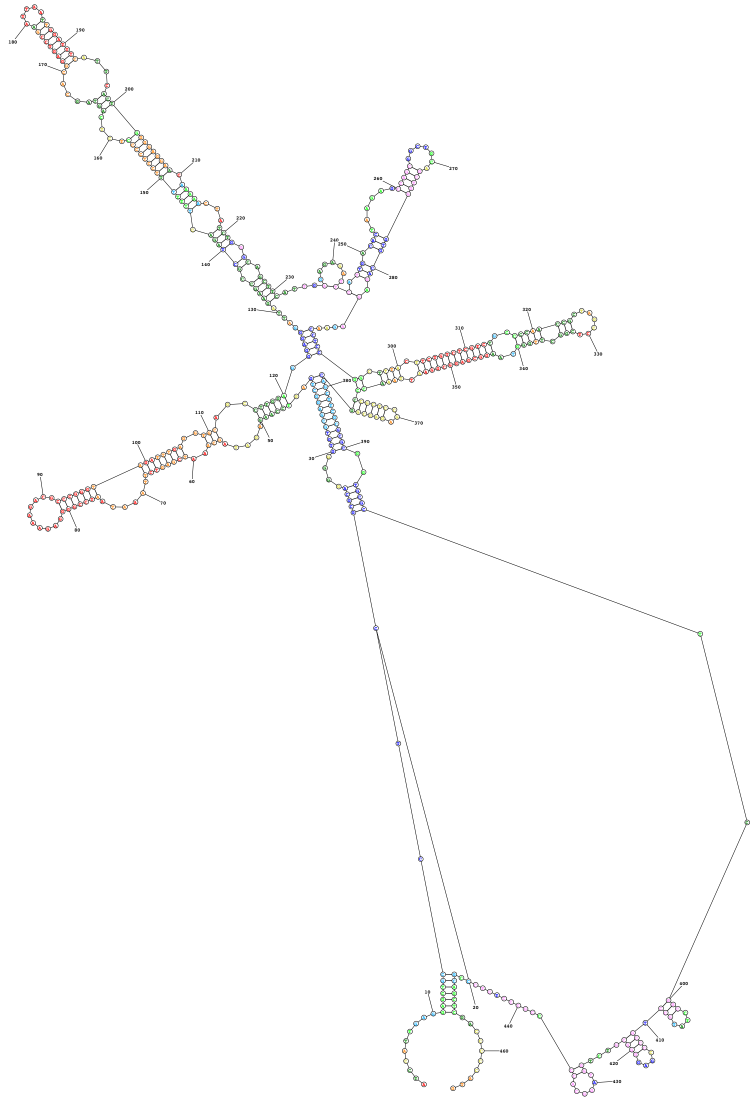

Probability >= 99%
99% > Probability >= 95%
95% > Probability >= 90%
90% > Probability >= 80%
80% > Probability >= 70%
70% > Probability >= 60%
60% > Probability >= 50%
50% > Probability
ENERGY = 36.5 DUSP6 NM_001946.4

B) *DUSP5*

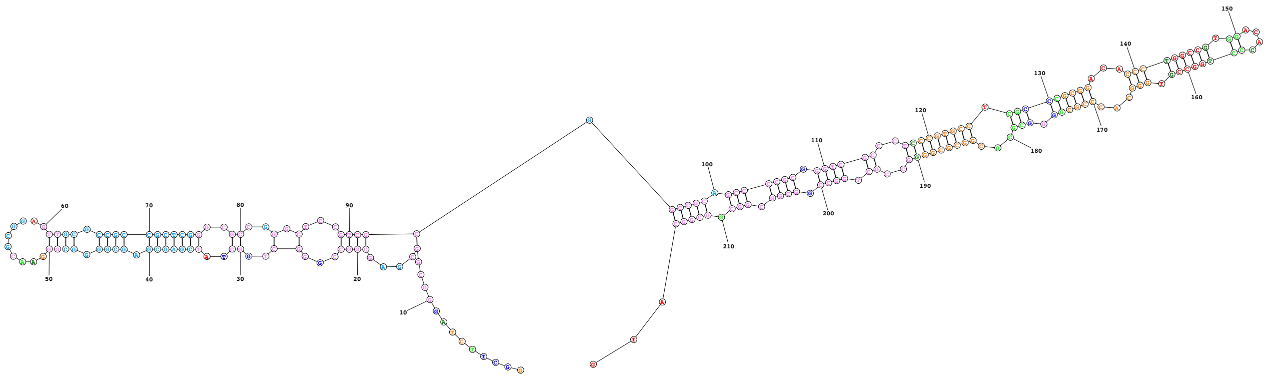

Probability >= 99%
99% > Probability >= 95%
95% > Probability >= 90%
90% > Probability >= 80%
80% > Probability >= 70%
70% > Probability >= 60%
60% > Probability >= 50%
50% > Probability

ENERGY = 14.0 DUSP5 NM_004419.4

C) *MYC*

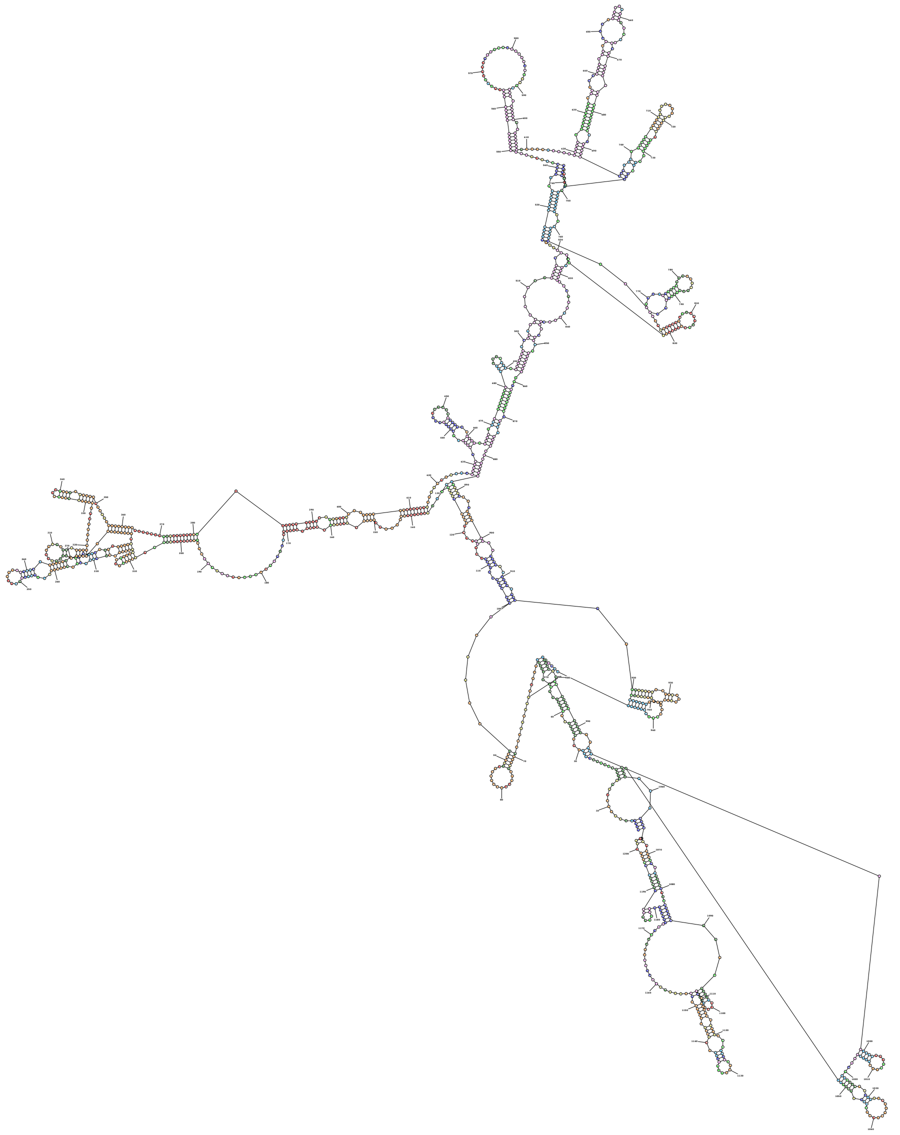

Probability >= 99%
99% > Probability >= 95%
95% > Probability >= 90%
90% > Probability >= 80%
80% > Probability >= 70%
70% > Probability >= 60%
60% > Probability >= 50%
50% > Probability

ENERGY = 91.1 MYC

D) *JUN*

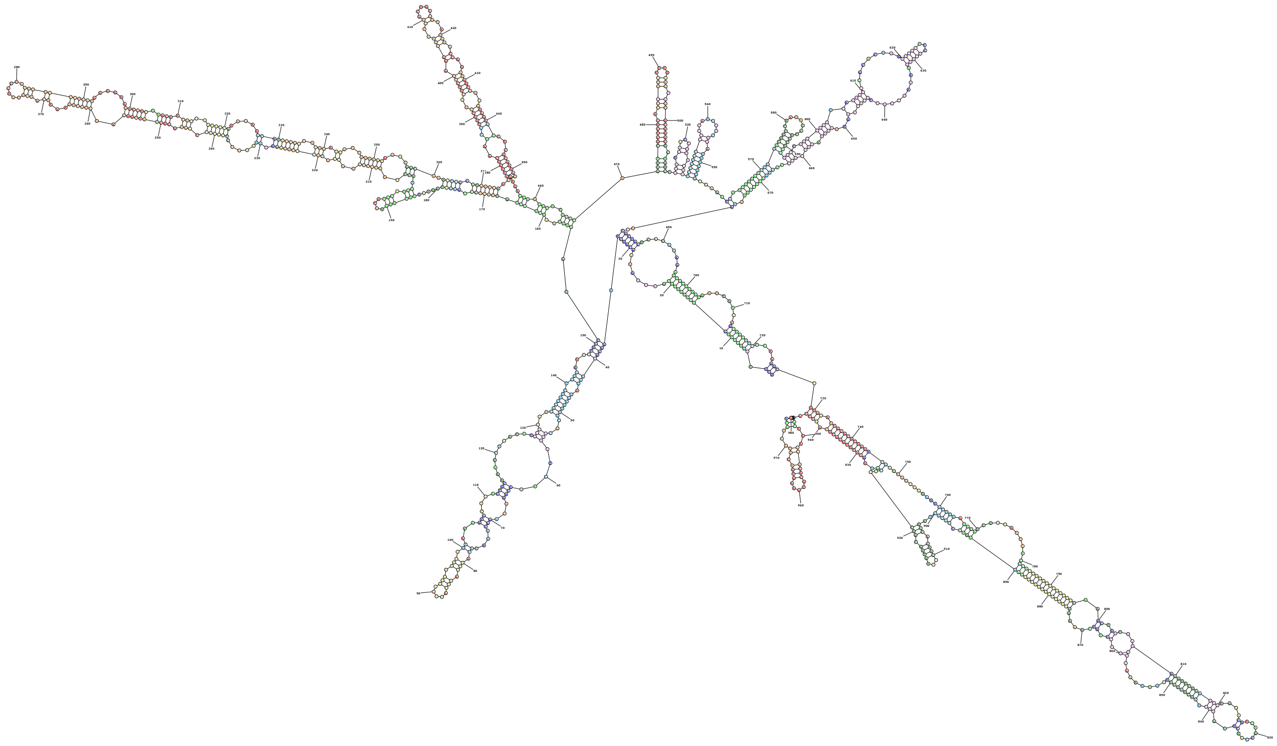

Probability >= 99%
99% > Probability >= 95%
95% > Probability >= 90%
90% > Probability >= 80%
80% > Probability >= 70%
70% > Probability >= 60%
60% > Probability >= 50%
50% > Probability

ENERGY = 77.7   Jun NM_002228.4

E) *FOS*

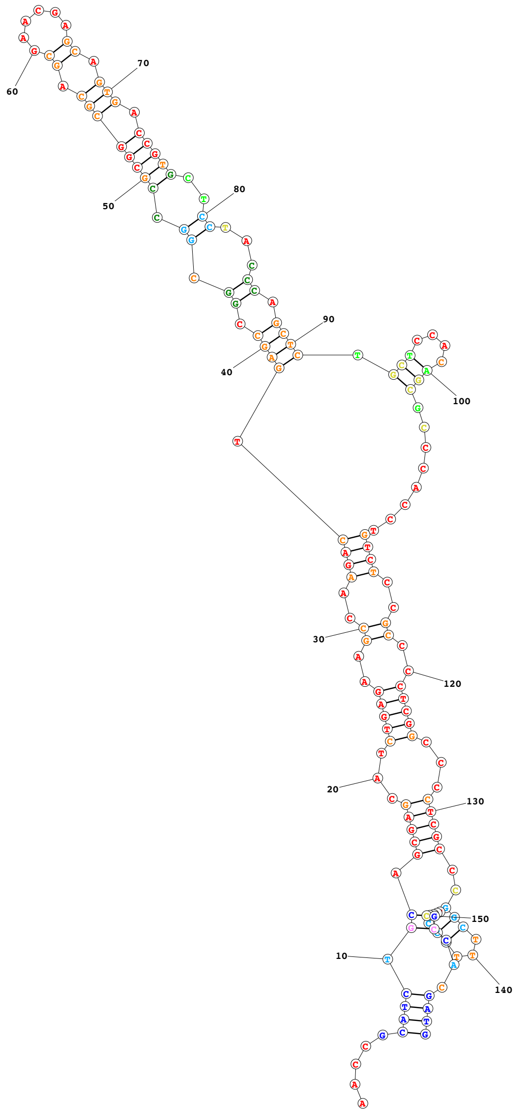

Probability >= 99%
99% > Probability >= 95%
95% > Probability >= 90%
90% > Probability >= 80%
80% > Probability >= 70%
70% > Probability >= 60%
60% > Probability >= 50%
50% > Probability

ENERGY = 14.2 FOS NM_005252.4

F) *EGR1*

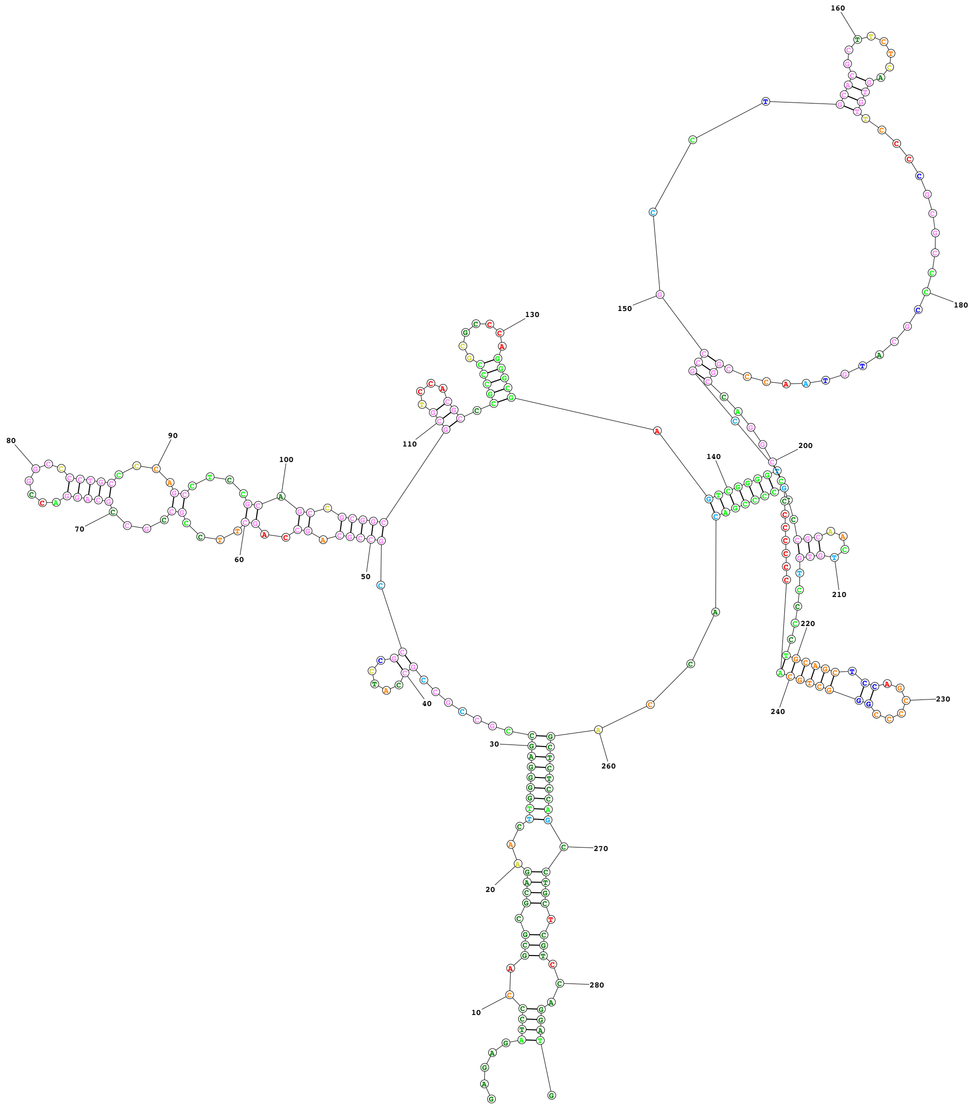

Probability >= 99%
99% > Probability >= 95%
95% > Probability >= 90%
90% > Probability >= 80%
80% > Probability >= 70%
70% > Probability >= 60%
60% > Probability >= 50%
50% > Probability

ENERGY = 19.7 EGR1 NM_001964.3

Figure S4: Secondary structure prediction in 5' UTRs of selected transcripts. Secondary structure predictions of 5'UTRs of human *DUSP6* **(A)**, *DUSP5* **(B)**, *MYC* **(C)**, *JUN* **(D)**, *FOS* **(E)**, and *EGR1* **(F)** transcripts were performed using the Predict a Secondary Structure Web Server v. 6.0.1 (https://rna.urmc.rochester.edu). The RNA Structure MaxExpect tool was used which generates structures composed of highly probable base pairs. **5' UTR sequences used for the prediction** (NCBI reference sequences, start codon is marked in yellow. Both CUG and AUG codons were reported to initiate the translation of the MYC transcript):

A) *DUSP6*

NM_001946.4, 5' UTR:

ATCCATTGAGGAGCTGCCTCGCGCAGGGGGTGTGCGAGGCTGAGTCCAAGAGATAGCAAATCGAGTCTTA
AATAATCCGGGGAGAAAGACGCCCGGGTAGATTTGAGGTGCAGCCTTGGAGGGAGGGATTAGAAGCCGCT
AGACTTTTTTTCCTCCCCTCTCAGTAGCACGGAGTCCGAATTAATTGGATTTCATTCACTGGGGAGGAAC
AAAAACTATCTGGGCAGCTTCATTGAGAGAGATTCATTGACACTAAGAGCCAGCGGCTGCAGCTGGGTGC
AGAGAGAACCTCCGGCTTTACTTCTGTCTCGTCTGCCCCAACCGCTAGCCTCGGCTTGGGTAAGGCGAGG
CGGAATTAAACCCCGCTCCGAGAGCGGCAGCTTCGCGCGCGGTGCGCTCGGCCTATGCCTGCCCCGAGGG
GCGTCTGGTAGGCACCCCGCCCTCTCCCGCAGCTCGACCCCCATG

B) *DUSP5*

NM_004419.4, **5' UTR:**

GGCTTCTAGGGCGGCGAGCGGCCGGGCTGGCTATCGAGCGAGCGGGGCGGGAACGCGGAGTTGCGCCGCC
GCTCGGGCGCCGGGCTCCGTCGCGGCCGCAGCCCCGCGGGTCGCCCTCCCGTGCCTCGCCCGCGGACACC
CTGGCCGTGGACACCCTGGCCGTGGGCACCCGCGGGGCGCGCGGCGCGGGGCCGCTGGCCGGCGGCGGCG
GCGGCATG

C) *MYC*

NM_001354870.1, 5' UTR:

GGAGTTTATTCATAACGCGCTCTCCAAGTATACGTGGCAATGCGTTGCTGGGTTATTTTAATCATTCTAG
GCATCGTTTTCCTCCTTATGCCTCTATCATTCCTCCCTATCTACACTAACATCCCACGCTCTGAACGCGC
GCCCATTAATACCCTTCTTTCCTCCACTCTCCCTGGGACTCTTGATCAAAGCGCGGCCCTTTCCCCAGCC
TTAGCGAGGCGCCCTGCAGCCTGGTACGCGCGTGGCGTGGCGGTGGGCGCGCAGTGCGTTCTCGGTGTGG
AGGGCAGCTGTTCCGCCTGCGATGATTTATACTCACAGGACAAGGATGCGGTTTGTCAAACAGTACTGCT
ACGGAGGAGCAGCAGAGAAAGGGAGAGGGTTTGAGAGGGAGCAAAAGAAAATGGTAGGCGCGCGTAGTTA
ATTCATGCGGCTCTCTTACTCTGTTTACATCCTAGAGCTAGAGTGCTCGGCTGCCCGGCTGAGTCTCCTC
CCCACCTTCCCCACCCTCCCCACCCTCCCCATAAGCGCCCCTCCCGGGTTCCCAAAGCAGAGGGCGTGGG
GGAAAAGAAAAAAGATCCTCTCTCGCTAATCTCCGCCCACCGGCCCTTTATAATGCGAGGGTCTGGACGG
CTGAGGACCCCCGAGCTGTGCTGCTCGCGGCCGCCACCGCCGGGCCCCGGCCGTCCCTGGCTCCCCTCCT
GCCTCGAGAAGGGCAGGGCTTCTCAGAGGCTTGGCGGGAAAAAGAACGGAGGGAGGGATCGCGCTGAGTA
TAAAAGCCGGTTTTCGGGGCTTTATCTAACTCGCTGTAGTAATTCCAGCGAGAGGCAGAGGGAGCGAGCG
GGCGGCCGGCTAGGGTGGAAGAGCCGGGCGAGCAGAGCTGCGCTGCGGGCGTCCTGGGAAGGGAGATCCG
GAGCGAATAGGGGGCTTCGCCTCTGGCCCAGCCCTCCCGCTGATCCCCCAGCCAGCGGTCCGCAACCCTT
GCCGCATCCACGAAACTTTGCCCATAGCAGCGGGCGGGCACTTTGCACTGGAACTTACAACACCCGAGCA
AGGACGCGACTCTCCCGACGCGGGGAGGCTATTCTGCCCATTTGGGGACACTTCCCCGCCGCTGCCAGGA
CCCGCTTCTCTGAAAGGCTCTCCTTGCAGCTGCTTAGACGCTGGATTTTTTTCGGGTAGTGGAAAACCAG
CCTCCCGCGACGATG

D) *JUN*

NM_002228.4, 5′ UTR:

GCTCAGAGTTGCACTGAGTGTGGCTGAAGCAGCGAGGCGGGAGTGGAGGTGCGCGGAGTCAGGCAGACAG
ACAGACACAGCCAGCCAGCCAGGTCGGCAGTATAGTCCGAACTGCAAATCTTATTTTCTTTTCACCTTCT
CTCTAACTGCCCAGAGCTAGCGCCTGTGGCTCCCGGGCTGGTGTTTCGGGAGTGTCCAGAGAGCCTGGTC
TCCAGCCGCCCCCGGGAGGAGAGCCCTGCTGCCCAGGCGCTGTTGACAGCGGCGGAAAGCAGCGGTACCC
ACGCGCCCGCCGGGGGAAGTCGGCGAGCGGCTGCAGCAGCAAAGAACTTTCCCGGCTGGGAGGACCGGAG
ACAAGTGGCAGAGTCCCGGAGCCAACTTTTGCAAGCCTTTCCTGCGTCTTAGGCTTCTCCACGGCGGTAA
AGACCAGAAGGCGGCGGAGAGCCACGCAAGAGAAGAAGGACGTGCGCTCAGCTTCGCTCGCACCGGTTGT
TGAACTTGGGCGAGCGCGAGCCGCGGCTGCCGGGCGCCCCCTCCCCCTAGCAGCGGAGGAGGGGACAAGT
CGTCGGAGTCCGGGCGGCCAAGACCCGCCGCCGGCCGGCCACTGCAGGGTCCGCACTGATCCGCTCCGCG
GGGAGAGCCGCTGCTCTGGGAAGTGAGTTCGCCTGCGGACTCCGAGGAACCGCTGCGCACGAAGAGCGCT
CAGTGAGTGACCGCGACTTTTCAAAGCCGGGTAGCGCGCGCGAGTCGACAAGTAAGAGTGCGGGAGGCAT
CTTAATTAACCCTGCGCTCCCTGGAGCGAGCTGGTGAGGAGGGCGCAGCGGGGACGACAGCCAGCGGGTG
CGTGCGCTCTTAGAGAAACTTTCCCTGTCAAAGGCTCCGGGGGGCGCGGGTGTCCCCCGCTTGCCACAGC
CCTGTTGCGGCCCCGAAACTTGTGCGCGCAGCCCAAACTAACCTCACGTGAAGTGACGGACTGTTCTATG

E) *FOS*

NM_005252.4, 5′ UTR:

AACCGCATCTGCAGCGAGCATCTGAGAAGCCAAGACTGAGCCGGCGGCCGCGGCGCAGCGAACGAGCAGT
GACCGTGCTCCTACCCAGCTCTGCTCCACAGCGCCCACCTGTCTCCGCCCCTCGGCCCCTCGCCCGGCTT
TGCCTAACCGCCACGATG

F) *EGR1*

NM_001964.3, 5′UTR:

GAGAGATCCCAGCGCGCAGAACTTGGGGAGCCGCCGCCGCCATCCGCCGCCGCAGCCAGCTTCCGCCGCC
GCAGGACCGGCCCCTGCCCCAGCCTCCGCAGCCGCGGCGCGTCCACGCCCGCCCGCGCCCAGGGCGAGTC
GGGGTCGCCGCCTGCACGCTTCTCAGTGTTCCCCGCGCCCCGCATGTAACCCGGCCAGGCCCCCGCAACT
GTGTCCCCTGCAGCTCCAGCCCCGGGCTGCACCCCCCCGCCCCGACACCAGCTCTCCAGCCTGCTCGTCC
AGGATG

Figure S5

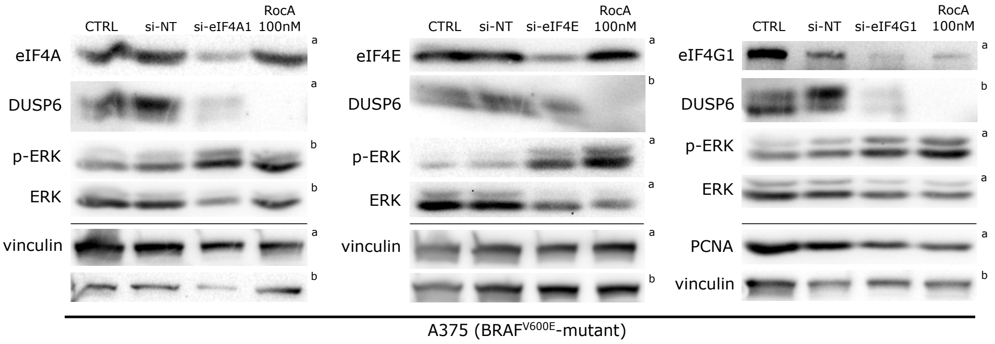

Figure S5: Impact of the knockdown of individual eIF4F subunits on DUSP6 expression and p-ERK levels in melanoma cells. Western blot analysis of A375 cells transiently transfected with siRNAs specific for eIF4A1, eIF4E, and eIF4G1 for 72h. Non-targeting siRNAs (si-NT) were transfected in parallel as negative controls. 20 h treatment with RocA served as a positive control, while the negative control samples (CTRL) were treated with an equivalent volume of the vehicle (DMSO). Vinculin and PCNA served as loading controls. The upper index letters refer to the corresponding loading control detected on the same membrane.

Figure S6

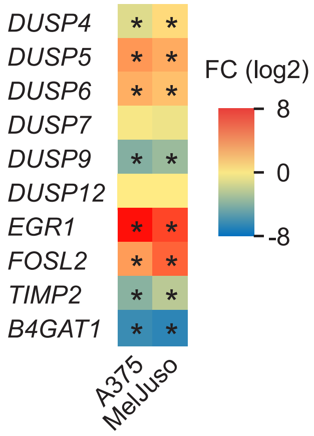

Figure S6: *DUSP* gene family mRNA expression heatmap. A375 and MelJuso cells were treated with 100 nM Rocaglamide A (RocA) or vehicle (DMSO) for 20 hours. Extracted RNA has been analyzed by RNA-seq. Obtained data were processed by DESeq2 and plotted as fold changes (FC). Significant expression changes in response to RocA ($q < 0.05$) are denoted with an asterisk. Examples of significantly upregulated (*EGR1* and *FOSL2*) and downregulated (*TIMP2*, *B4GAT1*) genes in response to RocA were included for comparison.

Figure S7

A

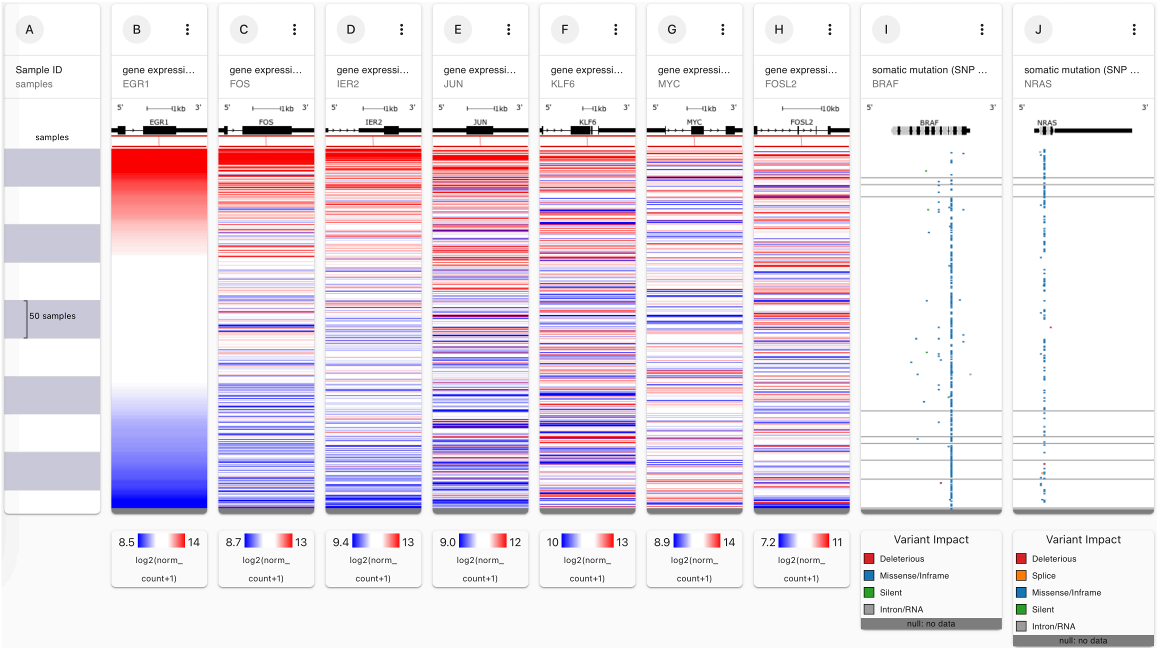

Sample ID
samples
samples
50 samples
gene expressi…
EGR1
FOS
IER2
JUN
KLF6
MYC
FOSL2
somatic mutation (SNP …
BRAF
NRAS
8.5
14
8.7
13
9.4
13
9.0
12
10
13
8.9
14
7.2
11
log2(norm_
count+1)
Variant Impact
Deleterious
Splice
Missense/Inframe
Silent
Intron/RNA
null: no data

B

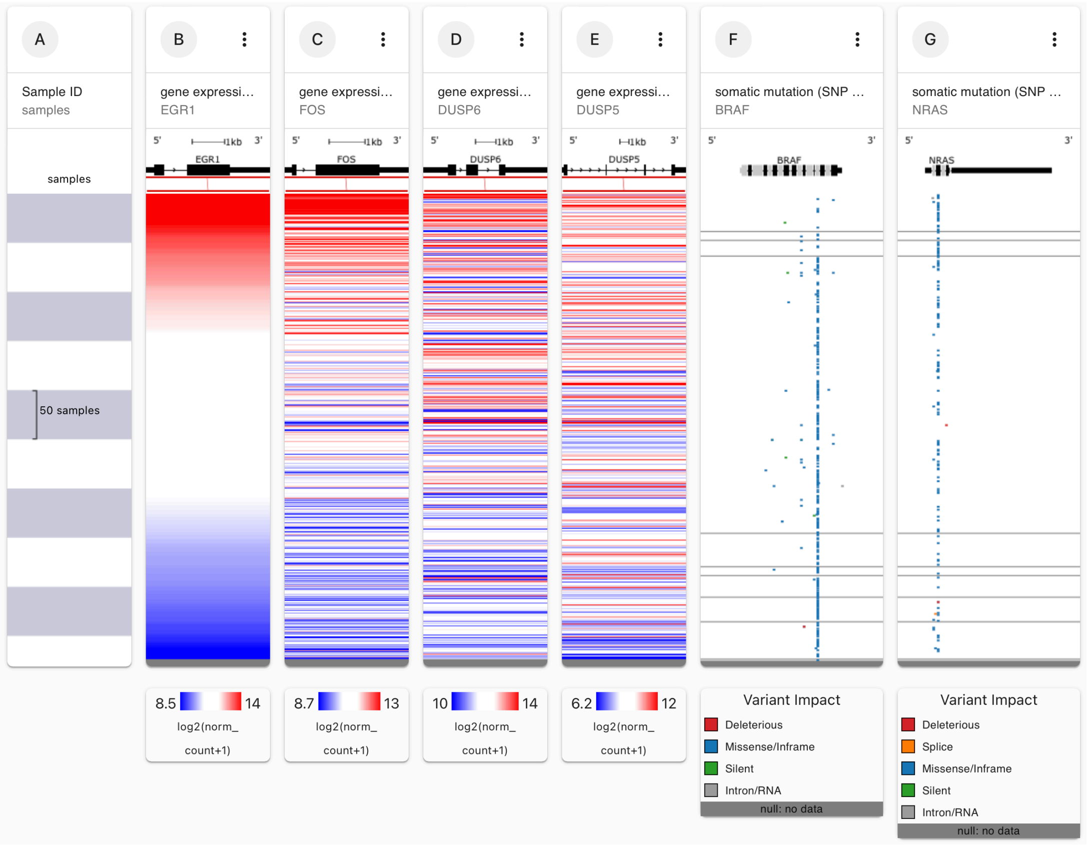

Sample ID
samples
samples
50 samples
gene expressi…
EGR1
FOS
DUSP6
DUSP5
somatic mutation (SNP …
BRAF
NRAS
8.5
14
8.7
13
10
14
6.2
12
log2(norm_
count+1)
Variant Impact
Deleterious
Splice
Missense/Inframe
Silent
Intron/RNA
null: no data

Figure S7: *EGR1* expression levels in patient tumor samples. Gene expression data generated by the TCGA Research Network (https://www.cancer.gov/tcga) were analyzed and visualized using the Xena Functional Genomics Explorer (https://xenabrowser.net) that allows users to explore functional genomic data sets for correlations between genomic and/or phenotypic variables. The *EGR1* gene expression in 481 human melanomas was compared to the expression of genes encoding transcription factors *FOS*, *IER2*, *JUN*, *KLF6*, *MYC*, and *FOSL2* **(A)**, the MAPK phosphatase genes *DUSP5*, and *DUSP6* **(B)**, and the mutational status of *BRAF* and *NRAS* oncogenes **(A, B)**.

Dataset S1: Proteomics data – Changes in MAPK targets in RocA-treated A375 melanoma cells. (A separate MS Excel file)

Dataset S2: Proteomics data – Changes in MAPK targets in RocA-treated MelJuso melanoma cells. (A separate MS Excel file)

Dataset S3: RNA-seq data – Differential gene expression in RocA-treated A375 melanoma cells. (A separate MS Excel file)

Dataset S4: RNA-seq data – Differential gene expression in RocA-treated MelJuso melanoma cells. (A separate MS Excel file)

Dataset S5: Ingenuity Pathway Analysis of RNA-seq data – Upstream Regulator prediction and Canonical Pathways activity analyses in A375 and MelJuso cell lines treated with either RocA or DMSO (vehicle) as described in the SI Materials and Methods section. (A separate MS Excel file)
